# Explanation of near-death experiences: a systematic analysis of case reports and qualitative research

**DOI:** 10.3389/fpsyg.2023.1048929

**Published:** 2023-04-20

**Authors:** Amirhossein Hashemi, Ali Akbar Oroojan, Maryam Rassouli, Hadis Ashrafizadeh

**Affiliations:** ^1^Student Research Committee, Nursing and Midwifery School, Ahvaz Jundishapur University of Medical Sciences, Ahvaz, Iran; ^2^Department of Physiology, School of Medicine, Dezful University of Medical Sciences, Dezful, Iran; ^3^Cancer Research Center, Shahid Beheshti University of Medical Sciences, Tehran, Iran; ^4^School of Nursing, Student Research Committee, Dezful University of Medical Sciences, Dezful, Iran

**Keywords:** near-death experience (NDE), out-of-body experience (OBE), death, systematic review, psychological experiences

## Abstract

**Background and objective:**

Some individuals report a near-death experience (NDE) after a life-threatening crisis, which refers to a range of subjective experiences related to impending death. This experience is a phenomenon with transcendental elements, which leads to deep permanent changes in both the individual and the social lives of the NDEr's. Therefore, this study aims to review the near-death experiences of individuals with different religious and cultural views.

**Methodology:**

This is a systematic analysis study. All the case report, case series and qualitative research studies which presented patients' NDE experiences were included in the study, without language restrictions, and in the period of 1980–2022. The stages of screening, selection, data extraction, and quality assessment have been gone through by two of the researchers. Data analysis and synthesis has been done qualitatively. JBI Critical Appraisal Checklist tool was used to evaluate the quality of the included studies.

**Findings:**

After the initial search, 2,407 studies were included, 54 of which underwent final examination. The total number of the NDEr's in the studies was 465 men, women, and children. Among these studies, 27 were case reports, 20 were case series, and 7 were qualitative studies. Near-death experiences have been categorized into 4 main categories and 19 subcategories. The main categories include emotional experiences (2 subcategories), cognitive experiences (4 subcategories), spiritual and religious experiences (4 subcategories), and supernatural experiences [9 subcategories in two categories (out of body experiences, and supernatural and metaphysical perceptions)].

**Conclusion:**

The most frequent near-death experiences were supernatural experiences, especially the experience of leaving the body. The basis and the content of the patterns mentioned by the NDEr's are similar, and the differences are in the explanation and the interpretation of the experience. There is a common core among them such as out-of-body experiences, passing through a tunnel, heightened senses, etc. Therefore, correct knowledge of near-death experiences leads to providing helpful answers to patients.

## Introduction

Near-death experiences (NDEs) are deep psychic, conscious, semi-conscious, or recollected experiences of someone who is approaching or has temporarily begun the process of dying which usually occur in life-threatening conditions (Greyson, 2007). In these experiences, the individual seems to be awake, and observes his/her body and the world from a point outside the physical body (Blanke et al., 2009). There are common features such as a feeling of inner peace, out of body experiences, traveling in a dark environment or “void” (usually associated with passing through a tunnel), reviewing one's life from childhood onwards, seeing a bright light, entering an extraterrestrial “other realm,” and communicating with “sentient beings” (Ring, 1980; Greyson, 1983; Moody, 2001; Martial et al., 2017). In a general classification, two factors have been introduced as the origin of these experiences. Van Lommel et al. (2001) and Hess (2019) differentiate between theories that link NDE to physiological changes in the brain and theories which see NDEs as a psychological reaction to approaching death (Van Lommel et al., 2001; Hess, 2019). The previous studies have highlighted the uniqueness of NDE memories in the autobiographical memory (Williams et al., 2008), stating that NDE memories contain more sensory, emotional, and self-referential details in comparison with the memories of other real and imaginary events, or the memories of a coma or impaired consciousness following an acquired brain dysfunction without NDE (Thonnard et al., 2013). Near-death experiences occur in various situations, including cardiac arrest in MI(myocardial infarction) (clinical death), the shocks caused by the blood loss after delivery or in postoperative complications, septic or anaphylactic shocks, electrocution, the coma caused by traumatic brain injury, intracerebral hemorrhage or cerebral infarction, suicide attempts, near drowning or suffocation experiences, apnea, and other cases where death is unavoidable (Van Lommel et al., 2001, 2017). The occurrence of near-death experiences is increasing thanks to improved survival rates through modern medical techniques. The results of a study show that sharing and investigating this phenomenon may happen 5–10 years after the occurrence of the experience, which often prevents the accurate evaluation of physiological and pharmacological factors (Van Lommel et al., 2001). In addition, the results of studies show that the prevalence of this phenomenon in the patients who have gone into cardiac arrest varies between 3.6 and 23% (Parnia et al., 2001; Schwaninger et al., 2002; Klemenc-Ketis et al., 2010). Other retrospective studies have estimated that between 43 and 48% of adults, and 85% of children who have been affected by life-threatening illnesses may have experienced the NDE phenomenon (Ring, 1980; Sabom, 1982; Morse, 2013).

The occurrence of these experiences leads to positive consequences in some NDEr such as a more altruistic life, higher spiritual growth, having interest in the meaning of life, fewer materialistic values, or a reduction in the fear of death (Noyes, 1980; Groth-Marnat and Summers, 1998; Knoblauch et al., 2001; Parnia et al., 2001; Moody, 2005; Khanna and Greyson, 2014a). Their subjective nature and the lack of a clear framework for these experiences make the description and the interpretation of these experiences dependent on individual, cultural, or religious factors (Van Lommel et al., 2017). Near-death experiences vary depending on the survivors' own cultural and religious background (Parnia, 2017), and are almost always described based on the individual's religious beliefs. Most of the early studies on NDEs only depict positive emotions (Ring, 1980, 1984).

Some studies have also mentioned negative experiences in NDEs, including “hellish” ones, although it seems that some NDEr's may still be reluctant to share their experiences (Charland-Verville et al., 2014; Cassol et al., 2019). Numerous quantitative and qualitative studies have been published on patients' experiences of this phenomenon. In the oldest study in this field, Raymond A. Moody compared the continental differences of experiencers (Moody, 2001, 2005; Schlieter and Schlieter, 2018). Combining research results allows qualitative studies to be conducted to reveal new insights or to identify whether subject saturation has occurred (Campbell et al., 2012). In addition to qualitative evidence, the majority of studies have collected quantitative data on patients' experiences through structured questionnaires or interviews. The results of the researches that have been carried out since 1981 in the field of NDE indicate that the treatment staff, especially nurses and doctors, have little knowledge of these experiences, while this knowledge is necessary to identify the NDEr's and help them cope with their experiences (Foster et al., 2009). As it was mentioned, these experiences cause deep and lasting changes in patients' personalities, which highlights the necessity of helping these patients to properly understand and perceive the NDE phenomenon and integrate its consequences (Foster et al., 2009; Van Lommel, 2010).

As far as the knowledge of the researchers allows, no systematic analysis has been designed in this field so far. Due to the fact that different studies have reached different results and the results of these studies have not been certain, so it is necessary to search for a definite result for a correct understanding of this phenomenon. In the present study, prior registration (Priori), data combination, more inclusive search based on the use of thesaurus systems MeSH and Emtree, investigation in large databases such as SCOPUS, WOS, MEDLINE/PubMed, Embase, Google scholar and ProQuest, use From Gray Literature, including: Thesis and conference papers and Proceedings, as well as the use of experts' opinions and the review of key journals, this systematic review can have a more comprehensive review of the relevant subject. Examining these experiences may pose challenges to the researchers of sciences such as psychology, parapsychology, psychiatry, medicine, philosophy of religion, and psychology of religion, each of which requires competent and well-reasoned answers. This systematic review reports a combination of the evidence related to patients' experiences- case reports, case series, and qualitative research- in order to achieve a comprehensive perception of patients' experiences. Considering the potential causes and the unpredictable aspect of this phenomenon, an overview of patients' experiences seems necessary.

## Research questions

This study focused on two specific review questions: (1) “What common experiences regarding the NDEr's accounts of NDE phenomena can be drawn from the results of the existing studies?”, and (2) “What broad knowledge can be gained from the NDEr's accounts of these common experiences?”

## Materials and methods

This systematic review has been prepared based on the Joanna Briggs Institute Reviewers' Manual (Mcarthur et al., 2015). Furthermore, the process of selecting the primary studies was done based on the PRISMA-P 2015 checklist (Institute of Medicine (US) Committee on Standards for Systematic Reviews of Comparative Effectiveness Research, 2011), and consensus-based clinical case reporting guideline development guidelines (Gagnier et al., 2013).

### Study eligibility criteria

#### Inclusion and exclusion criteria

##### Types of studies

In the current research, all case reports, case series, and qualitative research studies mentioning near-death experiences have been selected for entering. Other types of studies including cohort studies, case control, cross sectional, review, and clinical trial were not included in this research.

##### Types of participants

In this study, the eligible population included the individuals who had experienced unavoidable death and NDE without any age, gender, race, or ethnicity restrictions.

### Search strategy components

Without any language restrictions, studies were searched in PubMed/Medline, Scopus, Medline/Ovid, SPORTD (EBSCO), CENTRAL, and EMBASE, Cumulative Index to Nursing and Allied Health Literature (CINAHL), and Google Scholar search engines from Dec 15, 1980 to June 15, 2022. These were searched in ISI, Scopus, and ProQuest database. The details regarding the process of searching in the PubMed database have been provided below.

(“End Of Life”[tiab] OR End-Of-Life OR[tiab] “Determination of Death”[tiab] OR “Near-Death Experience”[tiab] OR “Out of body Experiences”[tiab] OR “Cardiac Death”[tiab] OR (Death[tiab] AND Cardiac[tiab])) OR “Sudden Cardiac Death”[tiab] OR (“Cardiac Death”[tiab] AND Sudden[tiab]) OR (Death[tiab] AND “Sudden Cardiac”[tiab]) OR “Cardiac Sudden Death”[tiab] OR (Death[tiab] AND “Cardiac Sudden”[tiab]) OR (“Sudden Death”[tiab] AND Cardiac[tiab]) OR “Sudden Cardiac Arrest”[tiab] OR (Arrest[tiab] AND “Sudden Cardiac”[tiab]) OR (“Cardiac Arrests”[tiab] AND Sudden[tiab]) OR (“Cardiac Arrest”[tiab] AND Sudden[tiab]) OR “Brain Death”[tiab] OR (Death[tiab] AND Brain[tiab]) OR “Brain Dead^*^”[tiab] OR “Coma Depasse”[tiab] OR “Irreversible Coma”[tiab] OR (Coma[tiab] AND Irreversible[tiab]) OR Coma^*^[tiab] OR Comatose[tiab] OR Pseudocoma^*^[tiab]) AND 1980/12/15:2022/12/15[dp].

The selection of keywords of this systematic review was done through a combination of Mesh Term, Free Text words, and Emtree. In case of coming across the studies in other languages such as Portuguese, Chinese, Japanese, etc. while searching, Google translation service was used, and for more certainty in this regard, a translator familiar with that language was asked for help. The aim of this study was to obtain all the articles that have been published in the field in order to minimize the risk of publication and reference bias in this article. Besides, PubMed's “My NCBI” (National Center for Biotechnology Information) email alert service was used to identify newly published studies. Manual search including gray literature, the reference list of the primary included studies, and key journals were searched to find more studies. If the researchers came across a study which matched the objectives of the present study, in case of not having access to the full text of the articles, data's being unpublished, or the existence of wrong and ambiguous data, the responsible author of the article would be emailed, and every 1–10 days, three other emails would be sent. The authors of the article were assured that the article would be reported appropriately. If no message was received from the author of the article after 3 emails, the article would inevitably be excluded. The two authors would try to reach an agreement in case of any disagreement, and in case of not reaching an agreement, the opinion of a third knowledgeable individual would be used as the decision criterion.

### Screening and selection

At first, the studies obtained in the search phase were transferred to the End Note software (× 7), and duplicate articles were removed from the software. Then two researchers (H, A and AH, H) separately reviewed all the primary studies based on the titles and the abstracts of the articles, and presented a number of studies which were in line with the search strategy in order to determine eligible studies based on the inclusion criteria. The selected studies were classified into three categories: relevant, irrelevant, and uncertain. The articles which were reported to be irrelevant by both researchers were excluded from the study, then the same two researchers separately evaluated the obtained studies based on the full texts of the articles. Each researcher provided a list of selected articles and the two lists were compared. In case of any disagreement between the researchers, it would be resolved through discussion and exchange of opinions. In case they could not reach a consensus, a third individual would act as an arbitrator. Then the agreement between the two arbitrators would be evaluated and, after a general agreement, the result would be reported as a statistical Kappa coefficient. According to this, in the present study, there was no disagreement between the two researchers in the steps performed. The agreement coefficient is calculated to be 100%.

### Study quality and the risk of bias assessment

The assessment of the risk of bias and the quality of study methodology was performed by two researchers (H, A and AH, H), separately, using JBI Critical Appraisal Checklists for Case Series and Case Reports, and JBI Critical Appraisal Checklist for Qualitative Research (Moola et al., 2020). These tools consist of 10 questions, and each question is answered in 4 ways: yes, no, unclear, and not applicable. Then all the studies were placed in three categories: Low Risk, High Risk, and Moderate Risk of Bias. The researchers tried to reach consensus in case of any disagreement.

Case Series and Case Reports checklist questions included 10 questions, respectively (1−Were there clear criteria for inclusion in the case series? 2−Was the condition measured in a standard, reliable way for all participants included in the case series? 3−Were valid methods used for identification of the condition for all participants included in the case series? 4−Did the case series have consecutive inclusion of participants? 5−Did the case series have complete inclusion of participants? 6−Was there clear reporting of the demographics of the participants in the study? 7−Was there clear reporting of clinical information of the participants? 8−Were the outcomes or follow up results of cases clearly reported? 9−Was there clear reporting of the presenting site(s)/clinic(s) demographic information? 10−Was statistical analysis appropriate.), and the questions of the qualitative checklist included 10 questions (1-Is there congruity between the stated philosophical perspective and the research methodology? 2−Is there congruity between the research methodology and the research question or objectives? 3−Is there congruity between the research methodology and the methods used to collect data? 4−Is there congruity between the research methodology and the representation and analysis of data? 5−Is there congruity between the research methodology and the interpretation of results? 6−Is there a statement locating the researcher culturally or theoretically? 7−Is the influence of the researcher on the research, and vice- versa, addressed? 8−Are participants, and their voices, adequately represented? 9−Is the research ethical according to current criteria or, for recent studies, and is there evidence of ethical approval by an appropriate body? 10- Do the conclusions drawn in the research report flow from the analysis, or interpretation, of the data?) (Moola et al., 2020).

### Data extraction

Data extraction was carried out by two researchers (H, A and AH, H), separately, using an information extraction form developed by the researcher. At first, an article was evaluated using this form as pilot evaluation, then it was used for evaluating other articles as well. Each researcher submitted the data extraction form of his articles and the two lists were compared. In case of any disagreement between the researchers, it would be resolved through discussion and exchange of opinions. If consensus was not reached, a third individual would act as an arbitrator, then the agreement between the two arbitrators would be evaluated. The following data be extracted from all studies: the first author's name, the article's year of publication, the country where the study had been done, the type of study design, the number of individuals who had experienced unavoidable death, and the characteristics including age (or age groups), gender, the NDEr's (near-death experiencers) underlying factors, and the type of NDE. The NDEr's quotes in the original studies were required for data analysis in order to preserve the meaning of the original text as a unit interpreted by the authors, or as raw data.

In order to analyze qualitative data, Graneheim and Lundman method was used (Graneheim and Lundman, 2004; Hsieh and Shannon, 2005). Semantic units were extracted from the participants' statements in the form of primary codes. The codes were also classified based on semantic and conceptual similarity and were as small and compressed as possible. There was a downward trend in data reduction in all analysis units and sub- and main classes. Finally, the data were placed in the main classes that were more general and conceptual, and the themes were abstracted. In addition, an example of data analysis has been shown in Table 1.

**Table 1:** An example of data analysis in the main themes.

**Theme**	**Categories**	**Primary code**	**Quotation**
Cognitive experiences	Changing the nature of time	° Belief in the power of God ° Long time ° The slowness of the speed of time ° Reasoning in actions and behaviors	It seems that time is in the hands of God's power. Apparently, it took me 4 min to die, but it took much, much longer for me there. The speed of time was slower than usual. Feeling more procrastination was associated with going deeper into the behaviors.
Emotional experiences	Positive experiences	° Absence of fear ° Feeling comfortable and relaxed	In the experiences I had, I was never accompanied by feelings like fear, terror and nightmare. I always felt a deep sense of peace and relief. Peace, comfort, rest and opportunity to be calm; Like a nice and pleasant holiday.
Spiritual and religious experiences	Meeting with dead	° Meet unknown people ° Introducing the unknown person to the experimenter ° Recounting the physical characteristics of the dead person to others after coming back to life	I had an aunt who died 20 years before I was born and I had no pictures or memories of her. In my experience I had a brief meeting with her and I was told that she is your aunt. He loved me a lot. After I returned to the world, when I described her appearance to my mother, she confirmed that she was my aunt.
Supernatural experiences	Out of body experiences	° The feeling of the soul not leaving the body ° Seeing the material body in an environment outside the physical body ° Near the ceiling	When I was taken out of the ambulance and being transferred to the operating room, I suddenly found myself lying on a stretcher in the corridor of the hospital. However, because of religious beliefs, I did not feel like I had died; because we believe in taking life and the difficulty of death and I did not experience anything like that. I was on top of my own body, but I didn't think that my soul was completely separated from my body. I remember that doctors and nurses were talking to each other. I even remember the color of their clothes. I really saw that I was near the ceiling and I was looking down.

## Results

### Descriptive characteristics of the articles

**Figure 1:**
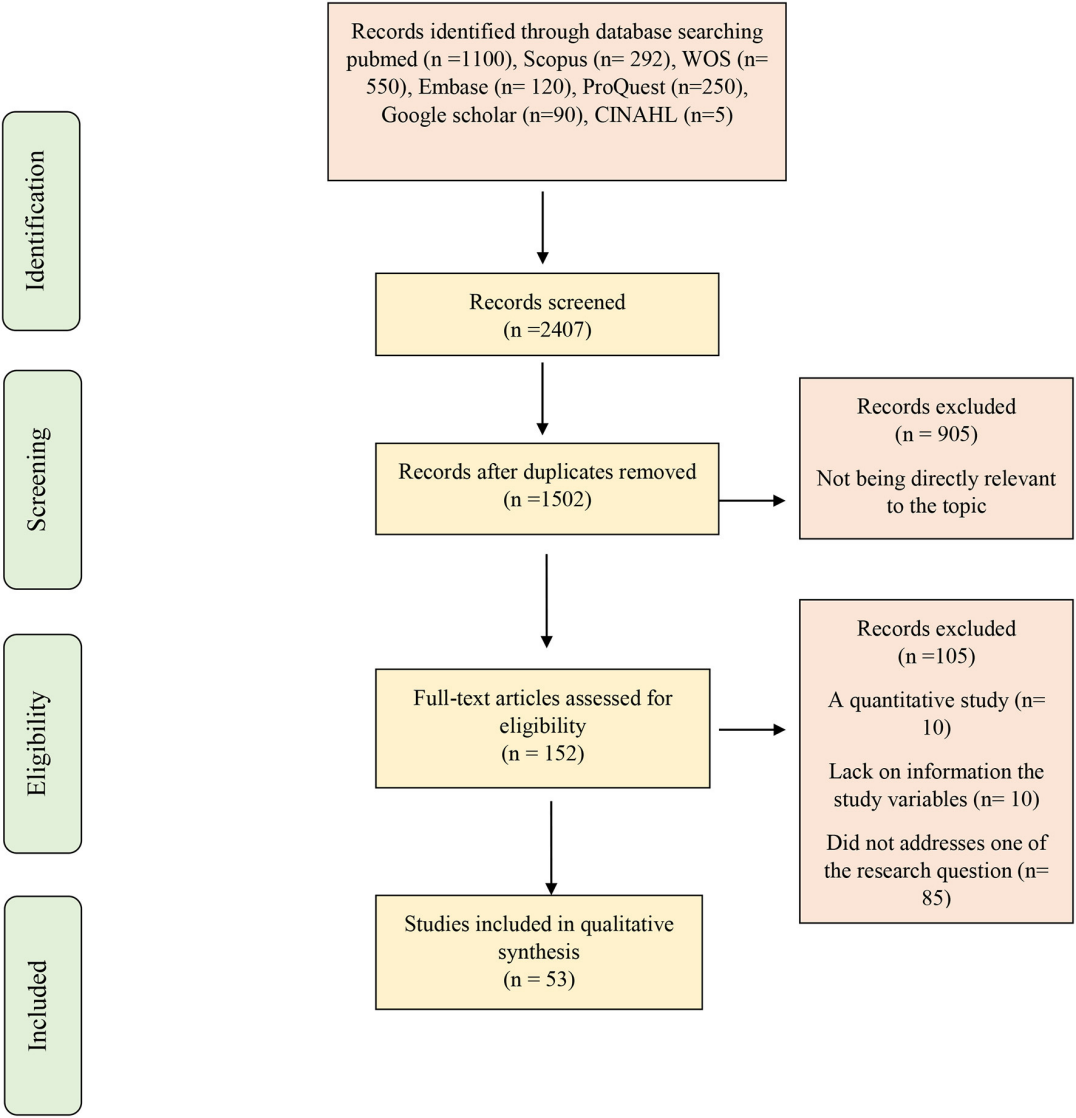
PRISMA schematic flowchart of enrolment and exclusions.

After searching, 2,407 articles were found. Using Endnote software, the titles and the abstracts of the articles were checked, and 905 duplicate articles were removed. Then the titles and the abstracts of 1,502 articles were examined by the researchers (H, A and AH, H). A total of 1,350 irrelevant articles were excluded based on the study objectives. At this stage, in case of doubting the relevance of an article with the study objectives, the full text of the article was reviewed by the researchers. In the next step, a search was done to access the full texts of the articles and, finally, the full text of 152 articles were reviewed. Considering the inclusion and the exclusion criteria based on the research objectives, some articles were excluded for the reasons given in the Prisma flowchart (Figure 1). To ensure that all the articles had been retrieved, the reference lists of the final articles were also manually searched; no studies were added in this stage. Finally, 54 studies were finalized.

Tables 2, 3 summarizes the characteristics of the included studies. In this table, the name of the first author, the title of the article, the year of publication, the NDEr's characteristics, the NDE's underlying factors, and after-death experiences are stated separately for each study. The studies had been published from 1985 to 2021. The 460 NDEr's included men (*n* = 126), women (*n* = 150), children (*n* = 22) and N/C (*n* = 162). The studies included 27 case reports, 20 case series, and 7 qualitative studies.

**Table 2:** Characteristics of the case report studies and background detail of near-death experiencers (*N* = 27).

**Number**	**References**	**Title of article**	**Age of the NDEr at the time of the NDE**	**Sex**	**Religious/cultural/social background**	**How long you had a near-death experience**	**Cause of NDEs**	**Experiences**
1	Morse (1983)	A near-death experience in a 7-year-old child	7	Child	The patient was from a deeply religious Mormon background	N/C	Drowning (com)	1. “Talking to the heavenly Father” 2. Remembrance statement 3. SAYING “it feels good to talk about it.” 4. Tunnel experience 5. Dark environment 6. Meeting strangers 7. Being in a new environment (paradise) 8. Meeting with the dead 9. The right to choose to return to earth 10. Reluctance to return to the material body
2	Abramovitch (1988)	An Israeli account of a near-death experience: s case study of cultural dissonance	N/C	Male	N/C	N/C	Heart attack	1. Increased alertness 2. Perception of darkness around 3. Feeling of falling 4. The feeling empty 5. Seeing yourself outside the body 6. Out-of-body experience 7. Trying to return to the material body 8. A sense of peace 9. Shining light 10. seeing others 11. Hearing voices 12. Lack of understanding of time 13. Communications with the dead
3	Irwin and Bramwell (1988)	The devil in heaven: a near-death experience with both positive and negative facets	N/C	Female	N//C	“I was told later it took about an hour to get me out. But I don't remember any of that.”	Accident	1. Out of body experience 2. MOVE up 3. Hearing voices 4. See the surroundings 5. Lack of a sense of calm 6. Tunnel experience 7. Seeing the light 8. Being in a new environment 9. See the church 10. Seeing people 11. Facing the devil
4	Pennachio (1988)	Near-death experiences and self-transformation	N/C	Female	Pregnant	Three consecutive near-death experiences within 4 weeks.	Deliberate drug overdose, resulted in a Cesarean section and brought on subsequent unconsciousness and cardiac arrest	**First trip** 1. Upon going into a coma, she felt as though she had gone to hell. 2. I half ran and half walked; it seemed like hours, but it must have been only seconds. I don't know how long. 3. My entire life appeared before me. 4. Then there was Christ and he told me it was not my time. 5. Everything was calm, no fear at all.
								**Second trip** 1. This encounter was unpleasant and unlike the first. It was frightening, devastating. 2. The faces of people were distorted. Some were laughing and others were screaming at me. 3. I felt that I should stay and sort things out. 4. I was convinced there was no eternal peace. 5. I was able to evaluate all of my friends. 6. They appeared to me as they actually were. 7. I knew who was and was not my friend. **Third trip** 1. I was out of my body and being filled with knowledge. 2. This knowledge led to great love and understanding of humanity. There was a sense of cramming, as if I was being crammed with knowledge and power. 3. You are training for the transformation which follows. 4. I was being turned completely around; I was being made over. 5. I was made different; I'm not the person I was.
5	Serdahely and Walker (1990)	A near-death experience at birth	At birth	Female	N/C	The patient at the age of 23 has recounted her experience	Wrapping the umbilical cord around the neck and being dead for 5 min	1. Travel through the tunnel to the light 2. No light entering 3. Described the NDE as frightening and distressing 4. Experience feelings of “powerlessness, anger, aggravation, and numbness.” 5. Not seeing your body 6. Trying to return to your physical body
6	Sutherland (1990)	Near-death experience by proxy: a case study	34	Male	He stated that he was not a religious or spiritual person before his experience	N/C	During the wake (in prison)	1. Tingling experience 2. Out-of-body experience 3. Seeing yourself from outside the body 4. Failure to understand the feeling of pain 5. Increasing awareness 6. See other people in the bubble 7. Understanding love 8. Oneness with the world 9. Change of attitude 10. Losing the fear of death
7	Serdahely and Walker (1990)	The near-death experience of a non-verbal person with congenital quadriplegia	8	Child	He had an NDE when he was two and a half years old, and because of his speech limitations, he had to wait almost 37 years to communicate about his childhood NDE.	He has indicated that he had four OBEs before he was 21 years old and three OBEs after that age.	High fever, increased spinal fluid pressure, and muscular contractions with increased spasticity	1. The feeling of relaxation and absence of pain 2. Out-of-body experience 3. Seeing yourself from outside the body 4. Awareness of events happening around 5. Lack of tunnel experience and darkness 6. Seeing the bright light at the end of the stairs 7. Listening to music 8. Understanding the presence of others 9. Change in the nature of time 10. Communication with God through telepathy 11. Inability to choose to return to the physical body 12. To see the future 13. Feeling of floating 14. Losing the fear of death 15. Move to the ceiling 16. Seeing the dead 17. Being in a new Environment (paradise)
8	Bhowmick (1991)	Recurrent near-death experience with post-vagotomy syndrome	67	Male	Caucasian cultural	NDE has happened nine times over the last 20 years	Post-vagotomy syndrome (hypoglycemia)	1. Out-of-body experience 2. Seeing your body from the outside 3. Move to the ceiling
9	Gómez-Jeria (1993)	A near-death experience among the Mapuche people	N/C	Male	N/C	N/C	Cataleptic-like state for 2 days	1. Meeting with the dead 2. Presence in new places (volcano) 3. Understanding the presence of others 4. Desire not to return 5. Awareness that the time of his death is not now 6. Meeting a man with a book
10	Bonenfant (2000)	A Near-Death Experience Followed by the Visitation of an “Angel-Like” Being	31	Female	A research manager for individuals with mentally retardation	Two minutes	Drowning in the swimming pool	1. The subject found herself slowly drifting upwards in a dark environment. 2. She was able to perceive depth within that darkness. 3. She witnessed a childhood scene involving her still-living younger sister. 4. While moving toward the light, the subject felt that she was passing through a dark tunnel, slowly at first, but later with great acceleration.
								5. As she moved within the light she was filled with awe, peace, and love. 6. The angelic being radiated a sense of “motherly love” to the subject. 7. The subject remarked that her only desire was to reach the safety of those outstretched hands. But when she was nearly within reach, the figure withdrew her hands and told the subject, through her eyes, that is was not yet her time and that she would have to return.
11	Kelly et al. (2000)	Can experiences near death furnish evidence of life after death?	12	Child	N/C	N/C	After being given ether for tonsillectomy	1. Going down a tunnel to a shining light 2. Sense of peace 3. out-of-body experience 4. Seeing yourself from outside the physical body 5. Move to the ceiling 6. Passing through walls 7. Trying to communicate with others
12	Kellehear (2001)	An Hawaiian near-death experience	N/C	Female	N/C	N/C	Cardiac arrest	1. Out of body experience 2. Seeing your body from the outside 3. Seeing others 4. Meeting with the dead 5. Being attracted to a certain place 6. Reluctance to return to life 7. Lack of discretion in staying or leaving the environment 8. Awareness that the time has not yet come
13	Green (2001)	The near-death experience as a shamanic initiation: a case study	40	Female	She was a Caucasian woman. Although as a child she had been raised in a strict Protestant church, she was not a particularly religious person.	N/C	Automobile accident	1. Exposure to light 2. Out-of-body experience 3. Tunnel experience 4. Sense of flight 5. Sense of fear 6. Understanding the feeling of being loved 7. Encountering the dead (having a guide) 8. Listening to music 9. Encounter with god 10. Review of life (Mary did not see her life, but all the prayers she has prayed so far) 11. Move to the ceiling 12. Seeing your body 13. Awareness of the surrounding environment
14	Bonenfant (2001)	A child's encounter with the devil: an unusual near-death experience with both blissful and frightening elements	6	Child	N/C	8 h	Automobile accident	1. Out-of-body experience 2. Observing the surrounding environment 3. Tunnel experience 4. Facing the devil and feeling terrified 5. Experience the bright light 6. Encountering the dead 7. The feeling of security caused by God's presence 8. Being in a new territory 9. Awareness of the presence of an angel
15	Brandt et al. (2005)	Out-of-body experience as possible seizure symptom in a patient with a right parietal lesion	44	N/C	N/C	N/C	Seizure	1. Feeling “lightheaded” 2. The feeling of “slowly going to the sky” 3. Seeing your body
16	Lopez et al. (2006)	Near-death experience in a boy undergoing uneventful elective surgery under general anesthesia	15	Child	N/C	N/C	Surgery (general anesthesia)	1. Out-of-body experience 2. Seeing your body from outside the body 3. The feeling of floating 4. Move to the ceiling 5. Feeling comfortable and relaxed 6. Tunnel experience 7. The idea that everything is real 8. Seeing the bright light at the end of the tunnel 9. Hearing voices 10. Involuntary return to the physical body
17	Brandt et al. (2009)	Out-of-body experience and auditory and visual hallucinations in a patient with cardiogenic syncope: the crucial role of cardiac event recorder in establishing the diagnosis	48	Male	N/C	N/C	Cardiogenic syncope	1. “He hallucinated many small-sized people (“like you see them on TV”) who were “marching like soldiers.” He could clearly hear their heavy footsteps.” 2. As he lay motionless, he fancied that he was standing upright and saw many children in winter clothes at his feet, screaming loudly and trying to tear him apart. again, the children he saw were smaller than real children. 3. “When he looked down, he could see his legs and feet. He did not see his sleeping body, but through a narrow field of vision he saw the real environment.”
18	Panditrao et al. (2010)	An unanticipated cardiac arrest and unusual post-resuscitation psycho-behavioral phenomena/ near death experience in a patient with pregnancy-induced hypertension and twin pregnancy undergoing elective lower segment cesarean section	24	Female	N/C	N/C	Cardiac arrest during childbirth	1. Remember to go to the operating room 2. Memories of “journey in dark lands” 3. Move toward the bright light 4. He could remember people whispering that he was “dead.” 5. Whenever he saw himself in the mirror, his vision focused on the eyes of his image and he felt that he was focusing on the image inside the eyes of his image. 6. He also felt as if he heard many people talking about something and someone telling him he was no more/he was dead. 7. Out-of-body experiences.
19	Cooper (2011)	Near-death experience and out of body phenomenon during torture-a case report	N/C	N/C	Black African The patient described himself as a Christian there was no overtly religious component to the experience.	N/C	Torture	1. Out-of-body experience 2. Upward movement of the soul 3. Perception of light and sound 4. Life review 5. After suffering 6. Familiarity with the future 7. Seeing yourself from outside the body 8. Seeing the light at the end of the tunnel 9. End of experience with the loss of consciousness 10. Altered sense of time
20	Facco and Agrillo (2012a)	Near-death-like experiences without life-threatening conditions or brain disorders: a hypothesis from a case report	N/C	N/C	The subject reported that he had rejected any form of religion by that time in his life, as a reaction to having gone to a catholic college for a year during his adolescence.	N/C	In waking state	1. Seeing white light 2. Empathetic fusion with the whole world 3. The feeling of love and happiness 4. Inability to describe in words 5. Overcome the fear of death 6. Complete loss of sense of time 7. Peace 8. A clear understanding of a reality beyond the ordinary world 9. Understanding everything about the world
21	Tassell-Matamua (2013)	Phenomenology of near-death experiences: an analysis of a Maori case study	N/C	Female	A Māori individual	N/C	Seriously ill	1. Out-of-body experience 2. Meeting with the dead 3. Encountering a spiritual being 4. Awareness of the procedures that have happened 5. Being in a new environment 6. The right to choose to return to earth 7. Losing the fear of death
22	Hausheer (2014)	Getting comfortable with near-death experiences. my unimaginable journey: a physician's near-death experience	20	Female	N/C	N/C	Guillain-Barre syndrome	1. Out-of-body experience 2. Looking at yourself from outside the body 3. Awareness of the procedures that have happened 4. Peace and tranquility 5. Understanding love 6. Seeing a bright light 7. Hearing voices 8. Losing the fear of death
23	Bos et al. (2016)	Out-of-body experience during awake craniotomy	50	Female	Non-religious	N/C	During an awake craniotomy	1. Out-of-body experiences with otoscopy 2. Floating 3. Move to the ceiling 4. Seeing your body outside the body 5. Awareness of events in the surrounding environment
24	Khanna et al. (2018)	Full neurological recovery from Escherichia coli meningitis associated with near-death experience	54	Male	In the study, it was mentioned that the race of the patient is white.	N/C	Deep coma because of *E. coli* meningitis	1. Awareness of events happening around the person 2. Out-of- body experience 3. Being in a dark plain 4. Seeing a bright light 5. Listening to music 6. Seeing colors 7. Understanding the presence of a woman 8. Love 9. Being in an infinite blackness 10. Meeting with the dead 11. Encountering a spiritual being 12. Communication without words 13. Not having the right to choose to return to the physical body
25	Hausheer (2020)	A physician's near-death experience	20	Male	Medical student	N/C	Respiratory arrest Resuscitation	1. Out of body experience 2. Move up 3. Seeing yourself from outside the body 4. Increase the field of vision 5. A sense of peace and tranquility 6. Seeing a bright light 7. Inability to fully describe events 8. Seeing the dark 9. Oneness with the environment 10. Seeing other souls 11. Being in a new environment 12. Increase awareness and knowledge 13. Ability to decide to return
26	Woollacott and Peyton (2021).	Verified account of near-death experience in a physician who survived cardiac arrest	32	Female	N/C	N/C	Cardiac arrest (during childbirth)	1. Increased alertness 2. The feeling of being real 3. Understanding what happened in the surrounding environment 4. Time stop 5. The emergence of a sense of calmness and peace 6. Sense of pleasure 7. A sense of harmony or unity with the universe 8. Seeing the light shine 9. Feeling sharper than usual 10. Out-of-body experience 11. Seeing an unearthly realm 12. Encountering a mystical being or presence or hearing an unidentifiable voice 13. The decision to return to life 14. Seeing yourself from outside the body 15. Spiritual transformation after NDE
27	Hosokawa et al. (2021)	Migraine with multiple visual symptoms and out-of-body experience may mimic epilepsy	18	Male	N/C	N/C	Epilepsy	1. Out-of-body experience 2. Seeing yourself from outside the body

**Table 3:** Characteristics of the case series and qualitative studies and background detail of near-death experiencers (*N* = 27).

**Number**	**References**	**Title of article**	**Number of cases**	**Age of the NDEr at the time of the NDE**	**Sex**	**Religious/cultural/ social background**	**Cause of NDEs**	**Experiences**
1	Lindley et al. (1981)	Near-death experiences in a Pacific Northwest American population: the Evergreen study. Anabiosis	55	N/C	31 female (62%) 19 men (34%) (5 non-experiencers not included)	The respondents were Caucasian. The proportion of non-religious and Protestant respondents was higher.	It was very different in the participants such as (accidents, suicide, and illness)	1. I felt very good and all the pain was just gone instantly. 2. Words commonly used to describe this stage are “peace,” “happiness,” “painlessness,” and “tranquility.” 3. Floating in the air about 5 feet above the end of the hospital bed. 4. Understanding all movements, people, and objects that could not be aware of their existence. 5. body-separation phase. 6. The awareness shifts from the external environment to the inner setting. 7. Some people find themselves moving through a void of blackness. 8. Travel through a long tunnel. 9. “Encountering the light.” This light is almost inevitably described as brilliantly white or golden. 10. Hellish and Negative Experiences. 11. The decision to return may be voluntary. However, the decision to return is not voluntary. Many reports fighting the return to their bodies. 12. Decision to return may involve a period of confusion and bargaining between the experience, the guide, and “God Himself.” 13. The Life Review.
2	Morse et al. (1985).	Near-death experiences in a pediatric population a preliminary report	8	16	Child	With devout Mormon parent	During arthrotomy and CPR	1. Sense of Peace 2. Moving to the top of a dark staircase 3. Decide to return
				6	Child	The parents follow traditional Christian beliefs, but are not members of any church, and there was no family teaching about the existence of the soul or the afterlife.	Cardiac arrest	1. Recalling memories during coma 2. Out-of-body experience 3. The experience of being suspended 4. Tunnel experience 5. Seeing a bright light 6. A sense of peace
				11	Child	Her family are devout Christians who attend church regularly and believe in the existence of a soul and an afterlife.	Cardiac arrest	1. Having a bad dream 2. Being blamed for doing something wrong
				8	Child	The patient attends the protestant Sunday school once a month	Hyperosmolar coma	1. Out-of-body experience 2. Feeling of floating 3. Seeing yourself from outside the body 4. Seeing the surroundings and doctors 5. Sense of fear
				4	Child	Not practicing Christian	Closed-head injury	N/C
				10	Child	N/C	Near-drowning survivor	N/C
				16	N/C	N/C	Cardiopulmonary arrest	N/C
				11	Child	Devout Christian with twice-weekly church attendance	N/C	N/C
3	Schorer (1985)	2 native American near-death experiences	2	N/C	N/C	N/C	Shot in battle	1. Out-of-body experience 2. Observing the mourning of those around you 3. Try to communicate with others 4. The decision to return to the material body 5. Encountering certain creatures
				N/C	N/C	N/C	N/C	1. Journey to Heaven 2. Meeting people 3. Failure to examine life
4	Herzog and Herrin (1985)	Near-death experiences in the very young	2	6-months	Child	N/C	Critical condition: renal failure and severe circulatory failure	1. Tunnel panic, a few months after his discharge 2. During the panic period, the patient talks fast, is unnecessarily scared, and overwhelmed 3. Expressing expressions that recognize death in relation to passing through the tunnel
				7	Child	N/C	Renal failure, seizure, cardiac arrest	1. Expression of near-death experience 2. Recalling the experience of cardiopulmonary resuscitation
5	Pasricha and Stevenson (1986)	Near-death experiences in India a preliminary report	4	10	Female	She had been a pious woman who read scripture that included a description of Yamraj	Paratyphoid disease	1. Meet others 2. Feeling tired 3. Meet Yamraj 4. An error in summoning the dead person
				30	Male	N/C	Typhoid	1. Meet others 2. See the new environment 3. Trying to escape 4. Damage by other people 5. An error in summoning the dead person 6. Forming a brand on the knee a few days after returning to life 7. Reinforcement of belief in God 8. To move against the will
				34	Male	N/C	Fever	1. Meeting people 2. Meeting with God 3. Reluctance to return 4. Becoming more honest after the NDE 5. To move against the will
				74	Male	N/C	None (during sleep)	1. Meeting people 2. To move against the will 3. Hearing voices 4. An error occurred in summoning a dead person 5. Severe burning sensation after regaining consciousness
6	Serdahely (1987)	The near-death experiences is the presence always the higher self	3	35-year-old woman had an NDE when she was 10 or 12 years old	Female	She is a member of the Catholic Church She doesn't speak French but interprets the experience through the use of a few, selects French phrases.	Child and sexual abuse	1. The departure of the soul from the body and displacement 2. Enter the darkness 3. Encountering the presence of a woman with a sense of motherhood 4. The feeling of unconditional love 5. A sense of calmness and guidance 6. The desire to return to the material body 7. A sense of mission 8. Out-of-body experience 9. Absence of any question in mind
				27-year-old woman explores an experience that happened to her when she was 5 years old	Female	She is a member of the Catholic Church. She doesn't speak French but interprets the experience through the use of a few, selects French phrases.	Child abuse	1. Out-of-body experience 2. Facing multiple people 3. Encountering the Virgin Mary who takes care of him 4. Understanding a mission on Earth
				N/C	Female	N/C	being molested	1. Out-of-body experience 2. The absence of another presence
7	Irwin (1987)	Out-of-body experiences in the blind	3	59	Female	N/C	Stroke	1. She reported once feeling very frustrated while watching television and then suddenly seeming to be walking on the window ledge
				90	Female	N/C	Degenerative condition	Once while in a “~stressful situation” she had the impression that “mind seemed to be elevated from the body.”
								No other details of the experience were recorded by the interviewer, and in particular it is not known if the experience had any visual content.
				56	Female	With a congenital deficit	Bleeding	1. Out- of- body experience. 2. She reported the impression of floating near the ceiling and looking down to see doctors working on her body. 3. The subject also had the feeling she could not leave through the ceiling and notes her exteriorized self repeatedly bumped into the ceiling. 4. She had the (not uncommon) reaction of scorn for her physical self during the experience. 5. More unusually, although she had been married for some 13 years at the time, she thought of herself by her maiden name. 6. The end of the OBE was marked by the experience of falling rapidly, then opening her eyes to find herself back in bed.
8	Serdahely (1990)	Pediatric near-death experiences	4	7	Child (boy)	N/C	He fell from a fishing bridge into a lagoon below	1. He floated out of his body. 2. He found himself in a dark, black tunnel. 3. He did not encounter any other spirits or presence while in the tunnel. 4. All of close relatives were alive at the time of his NDE. 5. He did not see a light at the end of the tunnel, but did see white clouds up above the tunnel. 6. When asked about a life review while in the tunnel, he said he did not have one. 7. When asked about time during his NDE, he replied: “~Time doesn't exist.”
				10	Child (girl)	N/C Her sister related that she had been clinically dead for ~30 s.	After recovering from spinal surgery heartbeat and respiration suddenly stopped	1. She said she felt “~peaceful,” “~relaxed,” and pain-free during her NDE. 2. Seeing a “whitish blue light” at the tunnel's end that drew her to it. 3. When asked if there was “~time” during her NDE, she said she was not sure whether or not time stood still.
				17	Child (girl)	N/C	An acute asthma attack and seizure	1. Suddenly, two “light figures” (her words) came to her. These beings were of the same bright light she saw at the tunnel's end. 2. As they were traveling down the tunnel, images from her past floated over her head. 3. The most memorable image was that of her father swinging her. Then saw her mother and how sad her mother and her Other relatives would be if she died. She felt “worried” for her family should she die. 4. She reported that it was difficult to judge time during her NDE. 5. She mentioned that the NDE “happened fast,” but that the light figures seemed to be going slowly.
				4	Child (boy)	N/C	He fell off a high diving board and landed on his head on the concrete below	1. Floating out of his body, he saw his mother below, holding him. He next found himself in a cloudiness or a fog. 2. Then a shaft of light that was bright, warm, and “yellow like the sun” penetrated the fog and surrounded him. 3. Out-of-body experience was at first scary, but then he felt he was with “~Triends” (his word), at which point he felt peaceful and pain-free. 4. A warm hand touched his shoulder as he looked at his body below, preventing him from turning around. A comforting, loving male voice, coming from the presence whose hand was on his shoulder, told him: “This is not your time. Do you want to go back or stay here?” 5. Who he thought might have been Jesus (he equivocate here).
9	Schnaper and Panitz (1990)	Near-death experiences: perception is reality	2	40	Female	N/C	N/C	She was on a stretcher in a corridor near an elevator and began to see “transparent” images of many people of all ages, in all sorts of garb. Regularly, groups would get on the elevator and leave, “like it was this day's toll of death.” She concluded that these people were dying and that because she was not put on the elevator, “my time had not come.”
								Felt incarcerated and was “always trying to escape” by pull in the needles out of her arms.
				30	Male	N/C	crushed chest	In a “dream” he had peed in bed and believed this was the reason people disliked him. He was unaware that the catheter was in place the entire time he was in the unit.
								In one of Mr. B.'s dreams, he had two brothers, Hercules and Colossus, who were wearing armor and protecting him. As he was about to be transferred from University Hospital, he heard the word “university” and thought about playing football with his brothers, perhaps on another university team. In fact, he had no brothers and his education was limited to the 11th grade. When questioned, he admitted that he had difficulty distinguishing reality from dreams, and strongly gave more credence to fantasy.
								In his sleep, he seemed to be looking at the TV screen. There he saw his wife wearing a black veil and his children standing in the cemetery. He could see the tombstone in front of them. His name was engraved on it. Remembering this dream, even at this later date, is still for Mr. B. It was extremely painful
10	Walker et al. (1991)	Three near-death experiences with premonitions of what could have been	3	A 35-year-old who had an NDE at the age of 4	Female	N/C	Extremely ill with influenza and bronchitis	1. Out-of-body experience 2. Move to the ceiling 3. The feeling of floating 4. To see the future 5. Sense of peace 6. Trying to get back into your body 7. Passing through walls 8. Absence of pain
				8- or 9-year-old who talks about her experience at the age of 30	Female	N/C	Feel of drowning	1. Seeing a bright light 2. The feeling of love 3. Understanding the presence of God 4. Understanding the presence of angels 5. Go fast on the road and stop at home 6. Seeing parents mourning next to their dead bodies
				17 and half years old who experience NDE at the age of 15	Female	N/C	Respiratory distress	1. Tunnel experience 2. Seeing a bright light 3. Understanding the friendly presence of others 4. Feeling of floating 5. Life review 6. To see the future 7. Observing the mourning of family members 8. Out-of-body experience 9. The feeling satisfied
11	Ring (1991)	Amazing grace: the near-death experience as a compensatory gift	5	N/C	Female	I was raised in a non-observant Conservative Jewish family in an overwhelmingly Jewish neighborhood in Philadelphia. I went through my teens as an atheist	During the wake after being discharged from the hospital due to the accident	1. Sense of peace 2. The feeling of floating 3. Move to the ceiling 4. Out-of-body experience 5. Seeing your body from outside the body 6. Seeing someone brilliant 7. Having a guide for the trip 8. Seeing religious people (Christ) 9. Seeing a bright light 10. Changing the nature of time 11. Tunnel experience 12. Being in a new environment 13. Increasing awareness 14. The feeling of love, peace and joy 15. Absence of pain
				N/C	Female	N/C	During the wake and mourning the loss of loved ones	When I was about six or so, I began to have the experience of dejavu 1. Out-of-body experience 2. The feeling of floating 3. Seeing a bright light 4. Communication with the dead 5. Seeing your body from outside the body
				35	Male	N/C	Overdose	1. Out-of-body experience 2. Move to the ceiling 3. Seeing your body from outside the body 4. Awareness of events happening around 5. Increasing awareness 6. Sense of peace 7. Seeing a bright light 8. Life review 9. Changing the nature of time
				N/C	Male	“A childhood faith in some sort of divine Father had been eroded by alcohol and materialism very early in life, and my logical mind would not accept what it could not rationalize.”	During surgery	1. Out-of-body experience 2. Tunnel experience 3. Seeing a bright light 4. The experience of flying at high speed 5. Hear the sound 6. Understand peace 7. Increase the power of sensory perception 8. Absence of pain 9. Understanding the presence of another 10. Being in a new environment 11. Seeing angels 12. Meeting with the dead 13. Having a travel guide 14. Lack of authority to return or stay 15. Changing the nature of time
				N/C	Male	He told that: “I don't believe in God, and I never go to church with its empty rituals and dogmas containing only vague and distorted reminiscences of the real thing.”	N/C	1. Seeing bright light 2. Understanding love 3. Understanding the presence of others 4. Life review 5. Understanding the feeling of freedom 6. Changing the nature of time 7. Understanding the presence of religious figures (God, Christ) 8. Increased awareness and alertness
12	Zhi-Ying and Jian-Xun (1992)	Near-death experiences among survivors of the 1976 Tangshan earthquake	81	Subjects' ages at the time of the earthquake averaged 31.4 years (S.D. = 11.3), with a range of 12–60 years. Four subjects (5 percent) were younger than 18 years old at that time; 51 (63%) were between 19 and 30 years old; and 26 (32 percent) were older than 30 years.	N/C	Of the 81 subjects, 79 (98 percent) were Han people and 2 (2 percent) were Moslems.	Earthquake	1. The feeling estranged from the body 2. Unusually vivid thoughts 3. Loss of emotions 4. Unusual bodily sensations 5. Life seeming like a dream 6. The feeling of dying 7. The feeling of peace or euphoria 8. The life review or “panoramic memory” 9. Thinking unusually fast 10. Time seeming to go faster than usual 11. An out-of-body experience 12. Sensation of the world being exterminated 13. A sense of weightlessness 14. One's self-feeling unreal 15. Senses unusually vivid
								16. Sudden understanding 17. Seeing deceased or religious figures 18. Thought, movement not under conscious control 19. The feeling of being pulled or squeezed 20. An unearthly realm of existence 21. Being controlled by an external force 22. Senses blurred or dull 23. Ambivalence about death 24. The feeling detached from one's surroundings 25. Being judged or held accountable 26. The world seeming unreal 27. Time seeming to slow down or stop 28. Visions of the future 29. The feeling of cosmic unity 30. Tunnel-like dark region 31. Thinking blurred or dull 32. A border or point of no return 33. An unnaturally brilliant light 34. A feeling of having been dead 35. Extrasensory perception (ESP) 36. Meaningful sounds 37. A feeling of joy or pleasantness 38. Meaningful visions 39. Feeling of being a different person 40. Unusual scents
13	Blackmore (1993)	Near-death experiences in india: they have tunnels too	5	50	Female	N/C	Concussion and loss of consciousness for a few moments	1. I was going through complete blackness. 2. There was a tingling sound of tiny bells in my ears. 3. The feeling was of complete relief and lightness. 4. It was not at all a feeling of deep slumber. 5. I said I was feeling ecstasy.
				72	Male	N/C	During angiography associated with a bypass operation	I suddenly got a feeling that someone was calling me away-almost saying “~now you have to go”- that your time “~here” is over.
								I cannot quite describe it-it was such a confused experience, but definitely I thought 4 or 5 figures were beckoning me to “go away” from this world.
				36	Male	He has had this near-death experience several times.	Severe palpitations twice came close to death	1. I seemed to be floating in a dark space. 2. I felt totally at peace.
				62	Male	N/C	He had a high fever and cough and was in a coma for 6–7 h.	1. He wrote it is so clearly imprinted on his subconscious mind that, even to this day, he can remember it as though it happened. 2. I was being flown away and up by two winged creatures (angels or fairies?) toward the higher skies. 3. It was an extremely exhilarating journey for my body and soul and I was fully enjoying the same.
				N/C	N/C	N/C	Accidentally electrocuted	I felt “~myself' light as a feather, shooting upwards at an indescribable speed-which can never be measured by the words “~speed” or “~time” and there, below me, above me, surrounding me on all sides were lights of all colors-shining spots which were not moving with me.
				63	Male		Cardiac arrest while being operated (liver abscess)	1. I traveled a few million miles away in an unknown space from the [operating] theater within a split of a second. 2. I was being loved, the love I had never experienced before, nor am experiencing after that event. 3. I feel I was beyond time and space.
14	Schoenbeck (1993)	Exploring the mystery of near-death experiences	4	N/C	Female	N/C	During awakening	1. Seeing things beyond the sight of others 2. Meeting the dead 3. Changing the nature of time 4. Awareness of impending death 5. Having a guide for the trip
				N/C	Female	N/C	Multiple myeloma	1. Meeting the dead 2. Awareness of impending death 3. Having a guide for the trip 4. See the tunnel 5. Ability to decide to return
				N/C	Female	N/C	Bone marrow transplant	1. Seeing a bright light 2. Seeing religious people (Christ) 3. Awareness of impending death 4. Out of body experience 5. Tunnel experience 6. Ability to decide to return
				N/C	Female	N/C	Intubation	1. The experience of being suspended 2. Awareness of the environment outside the hospital room
15	Greyson (1997)	The near-death experience as a focus of clinical attention	4	26-years old who had NDE 20 years before	Female	N/C	Near-drowning	1. Out-of-body experience 2. Tunnel experience 3. Encountering non-physical beings and communicating with them 4. Increasing awareness and science 5. Awareness of the future 6. Returning to life against the inner desire
				A 24-year-old who experienced an NDE at age 17	Male	N/C	Near-drowning	1. Hearing the cries and screams of tormented souls while visiting the site of the former Nazi concentration camp in Germany 2. Life review 3. Seeing scenes from the future
				14	Child	N/C	Cardiopulmonary resuscitation after electrocution	1. An NDE with a view of heaven and hell 2. Meeting with Christ 3. Meeting with the dead 4. Meeting with evil being
				30	Male	N/C	Post-operating hemorrhage	1. Sense of unconditional love
16	Ring and Cooper (1997)	Near-death and out-of-body experiences in the blind: a study of apparent eyeless vision	31	Ages ranged from 22 to 70 years	20 Female 11 male	They were all Caucasian, overwhelmingly Christian with respect to their original religious tradition, but varied greatly regarding their educational attainment and occupation.	Of our NDErs, 13 had their experience in connection with an illness or a surgical procedure; six as a result of an accident, usually involving an automobile; two were mugged; one was nearly killed by being raped; one almost perished in combat; and one survived a suicide attempt. (The totals here are 24 experiences since three persons had two separate NDEs each and were therefore counted twice in these tabulations.)	1. Feelings of great peace and wellbeing that attend the experience. 2. The sense of separation from the physical body. 3. The experience of traveling through a tunnel or dark space. 4. The encounter with the light. 4. The life review 5. Being in a place full of light and full of love (everything was from love and reflected love) 6. Meeting with Christ and asking the experiencer to return to life, but not accepting the request, and then she returned to her body by force and with pain and severity. 7. Hearing noise or music, in seven 8. Seeing one's own physical body 9. Meeting others, such as spirits, angels, or religious personages 10. Seeing a radiant light
17	Murphy (2001)	Near-death experiences in Thailand	10	N/C	N/C	N/C	N/C	1. Seeing religious figures 2. Meeting with the dead 3. Out-of-body experience 4. Tunnel experience 5. Being in a new environment 6. Inappropriate time to die
18	Knoblauch et al. (2001)	Different kinds of near-death experience: a Report on a survey of near-death experiences in Germany	82	The average age of the experiencers was 36 years	41 men and 41 women had NDE	N/C	Less than 50 percent of the respondents claimed to have been in a life-threatening situation when experiencing an NDE, and only 6 percent claimed to have been clinically dead.	50 percent of the NDErs reported positive emotions, and 43 percent had negative emotions. 1. Full mental awareness 2. Wonderful feelings 3. Entering another realm 4. Horrible feelings 5. Life review 6. Light 7. Tunnel experience 8. Heavenly realm 9. Met living persons 10. Out-of-body experience 11. The feeling of being dead 12. Contact with dead 13. Horrible realm 14. Met non-human beings
19	Van Lommel et al. (2001)	Near-death experience in survivors of cardiac arrest: a prospective study in the Netherlands	62	N/C	N/C	N/C	Resuscitation	1. Awareness of being dead 2. Positive emotions 3. Out-of-body experience 4. Moving through a tunnel 5. Communication with light 6. Observation of colors 7. Observation of a celestial landscape 8. Meeting with Deceased persons 9. Life review 10. Presence of border
20	Pasricha (1993)	Near-death experiences in South India: a Systematic survey	16	The median age of the subjects at the time of the NDE was 43.5 years (range 9-97 years) and it was 75 years (range 38-108 years) at the time of our first interview with them; the median time lapse between the NDE and the first interview was 20 years (range 2-70 years).	11 females 5 men	N/C	Seven subjects were reported to have been healthy prior to the NDE while nine (56%) subjects were suffering from a mild to severe physical illness prior to the experience. high- or low-grade fever (4 subjects), dysentery (2 subjects), typhoid, cough and asthma, and fits of unconsciousness (1 subject each).	1. Presence in new environments 2. Seeing your physical body 3. Seeing a man who had a book containing a list of deeds or mistakes 4. Review of life 5. Being brought back to life by mistake 6. Meeting with the dead 7. Repatriation from other territories by guides 8. The power of choice in returning to the material body 9. Sent by a loved one or an unknown face, but not because of a mistake 10. Residual symptoms in the physical body after an NDE 11. Changing attitude toward death
21	Purkayastha and Mukherjee (2012)	Three cases of near-death experience: is it physiology, physics or philosophy?	3	30	Female	Hindu, married with a 5-month-old baby	With severe head injury	1. Seeing a bright light 2. Floating 3. Seeing the new environment (paradise) 4. Telling what happened around him during his coma 5. Reluctance to return to the material body
				22	Male	Hindu	Cardiac arrest for around 10 minutes (anaphylactic shock)	1. Travel through a tunnel of white light 2. Sense of peace 3. Out-of-body experience 4. Observing what happened around him
				4	Child	Hindu	hypotensive shock	1. The experience of being in white and silver clouds 2. Time dilation
22	Ghasemiannejad et al. (2014)	Iranian Shiite Muslim near-death experiences: features and aftereffects including dispositional gratitude	20	Five of them were in 18–30 years old range, 14 of them were in 31–50 years old range and one of them was more than fifty	14 men and 6 women	Moslem	Serious illness (2), accident-related injury (8), childbirth (1), cardiac arrest (6), suicide attempt (2), not specified (1)	1. Passing in or through a tunnel 2. Positive emotions or feelings 3. Exposure to extraterrestrial light 4. Dealing with other beings and understanding the presence of others 5. Meeting with mystical beings 6. Meeting with the dead 7. A sense of time and place change 8. Life review 9. Encountering otherworldly realms 10. awareness of the surrounding environment 11. Viewing the environment up close to the ceiling 12. Sense of peace 13. The feeling of love 14. Sensory stimulation (seeing colors) 15. The occurrence of errors and the right time for death
23	Ghasemiannejad-Jahromi et al. (2018)	The investigation of near-death experiences, and the necessity of awareness about its elements	10	Average age of 38 years	2 women and 8 men	Moslem	Not stated	1. Changing the nature of time 2. Life review 3. Aggravation of the senses 4. Reaching a specific insight 5. Meeting with the dead and spiritual and religious figures 6. A feeling of oneness with existence 7. Observing the inner workings 8. Positive and pleasant emotions 9. Unpleasant and scary emotions 10. Leaving the body 11. Transcendental perception
24	Thomas (2020)	A near-death experience: a surgeon's validation	2	N/C	Female	N/C	During surgery	1. Out-of-body experience 2. Move to the ceiling 3. Seeing yourself outside the body 4. Awareness of what happened in the surrounding environment 5. Passing through walls 6. Dark environment 7. Seeing a bright light 8. Seeing the presence of others 9. Meeting with the dead 10. Seeing a new environment 11. Life review 12. awareness of the future 13. The ability to decide to return to the material body 14. Reluctance to return 15. A sense of love and peace
				N/C	Female	N/C	Spiritual crisis	1. Incompleteness of the article, not receiving more information 2. Out-of-body experiences 3. looking at the body from above
							Psycho-spiritual transformation	
25	Panagore (2020)	My deaths direct my life: living with the near-death experience	2	Second NDE occurred 35 years after the first experience	Male	N/C	1. Hypothermia 2. Blockage in left anterior descending artery	1. Change in the nature of time 2. Increased awareness and alertness 3. Facing a new environment (paradise) 4. Sense of satisfaction 5. Tunnel experience 6. Seeing a bright light 7. A sense of unity 8. Telepathy 9. The ability to decide to return 10. To see the future 11. Re-seeing the angel of death that he saw in the first experience 12. Tunnel experience 13. The power to decide to return to the physical body
				Second near-death experience was in 2016	Female	N/C	Struck by lightning during awakening	1. Hearing a voice saying, “You can't go now.” You have to go back to the children (I could see my two little children in my eyes.) You have to move your legs back and forth, back and forth, to keep the “fire” from reaching your heart. 2. Understanding all surrounding conversations 3. Out-of-body experiences 4. Seeing a very large white ball of shining light
26	Boado et al. (2020)	A case study on near death experience, its perceived effects and coping mechanisms: input to psychological and emotional adjustment training program	2	The respondents' age during the conduct of the study ranges from 50 to 65 years old	Male	They came from different locations.	N/C	1. Seeing a bright light 2. Meeting with the dead 3. One of the respondents felt peace in his heart 4. Tunnel experience in one of the participants
27	Khoshab et al. (2020)	Near-death experience among Iranian Muslim cardiopulmonary resuscitation survivors	8	N/C	Five males and three females.	N/C	CPR	1. A sense of lightness and flight 2. Tunnel experience 3. Seeing the light 4. Feeling satisfied 5. Out-of-body experience 6. Awareness of surrounding events 7. Changing the nature of time 8. Life review 9. Understanding pleasure 10. The experience of darkness 11. Losing the fear of death 12. Free movement of the soul 13. Hearing voices 14. Divine experiences about the Qur'an 15. Seeing and communicating with the saints

### Types of participants

**Figure 2:**
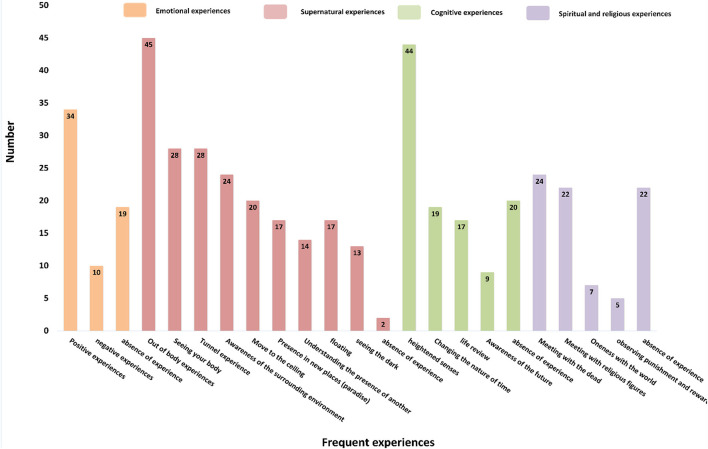
Categorization of individuals who had experienced unavoidable death.

Near-death experiences have been classified into 4 main categories, and 19 sub-categories. The main categories include emotional experiences (2 subcategories), cognitive experiences (4 subcategories), spiritual and religious experiences (4 subcategories), and supernatural experiences (9 subcategories in two categories (out of body experiences, and supernatural and metaphysical perceptions). The Individuals reported heightened senses, in 39 studies and out-of-body experiences, in 35 studies. In 28 studies, the patients reported positive experiences including love, the feeling of peace, and tranquility, and in 6 studies, they reported negative experiences, mostly torture and hellish experiences. Most of the experiences presented by the NDEr's were supernatural and metaphysical experiences, which are shown in Figure 2 and Table 4.

**Table 4:** The presence or absence of any category of experience in the included studies.

**References**	**Article**	**Emotional experiences**	**Supernatural experiences**	**Cognitive experiences**	**Spiritual and religious experiences**
Morse (1983)	A near-death experience in a 7-year-old child	**+**	**+**		**+**
Abramovitch (1988)	An Israeli account of a near-death experience: a case study of cultural dissonance		**+**	**+**	
Irwin and Bramwell (1988)	The devil in heaven: a near-death experience with both positive and negative facets	**+**	**+**	**+**	**+**
Pennachio (1988)	Near-death experiences and self-transformation	**+**	**+**	**+**	**+**
Serdahely and Walker (1990)	A near-death experience at birth	**+**	**+**		
(Sutherland (1990)	Near-death experience by proxy: a case study	**+**	**+**		**+**
Serdahely and Walker (1990)	The near-death experience of a non-verbal person with congenital quadriplegia	**+**	**+**	**+**	**+**
Bhowmick (1991)	Recurrent near-death experience with post-vagotomy syndrome		**+**		
Gómez-Jeria (1993)	A near-death experience among the Mapuche people				**+**
Bonenfant (2000)	A near-death experience followed by the visitation of an “angel-like” being	**+**	**+**	**+**	**+**
Kelly et al. (2000)	Can experiences near death furnish evidence of life after death?		**+**		
Kellehear (2001)	A Hawaiian near-death experience		**+**		**+**
Green (2001)	The near-death experience as a shamanic initiation: a case study	**+**	**+**	**+**	**+**
Bonenfant (2001)	A child's encounter with the devil: an unusual near-death experience with both blissful and frightening elements	**+**	**+**	**+**	**+**
Brandt et al. (2005)	Out-of-body experience as possible seizure symptom in a patient with a right parietal lesion		**+**		
Lopez et al. (2006)	Near-death experience in a boy undergoing uneventful elective surgery under general anesthesia	**+**	**+**	**+**	
Brandt et al. (2009)	Out-of-body experience and auditory and visual hallucinations in a patient with cardiogenic syncope: crucial role of cardiac event recorder in establishing the diagnosis		**+**		
Panditrao et al. (2010)	An unanticipated cardiac arrest and unusual post resuscitation psycho-behavioral phenomena/ near death experience in a patient with pregnancy induced hypertension and twin pregnancy undergoing elective lower segment cesarean section		**+**	**+**	
Cooper (2011)	Near-death experience and out of body phenomenon during torture—a case report		**+**	**+**	
Facco and Agrillo (2012a)	Near-death-like experiences without life-threatening conditions or brain disorders: a hypothesis from a case report	**+**		**+**	**+**
Tassell-Matamua (2013)	Phenomenology of near-death experiences: an analysis of a Maori case study	**+**	**+**		**+**
Hausheer (2014)	Getting comfortable with near-death experiences. My unimaginable journey: a physician's near-death experience	**+**	**+**	**+**	
Bos et al. (2016)	Out-of-body experience during awake craniotomy		**+**		
Khanna et al. (2018)	Full neurological recovery from Escherichia coli meningitis associated with near-death experience	**+**	**+**	**+**	**+**
Hausheer (2020)	A physician's near-death experience	**+**	**+**	**+**	**+**
Woollacott and Peyton (2021).	Verified account of near-death experience in a physician who survived cardiac arrest	**+**	**+**	**+**	**+**
Hosokawa et al. (2021)	Migraine with multiple visual symptoms and out-of-body experience may mimic epilepsy		**+**		
Lindley et al. (1981)	Near-death experiences in a Pacific Northwest American population: the evergreen study. Anabiosis	**+**	**+**	**+**	
Morse et al. (1985)	Near-death experiences in a pediatric population a preliminary report	**+**	**+**		
Schorer (1985)	2 native American near-death experiences		**+**		**+**
Herzog and Herrin (1985)	Near-death experiences in the very young		**+**		
Pasricha and Stevenson (1986)	Near-death experiences in India a preliminary report		**+**	**+**	**+**
Serdahely (1987)	The near-death experiences is the presence always the higher self	**+**	**+**		**+**
Irwin (1987)	Out-of-body experiences in the blind		**+**		
Serdahely (1990)	Pediatric near-death experiences	**+**	**+**	**+**	**+**
Schnaper and Panitz (1990)	Near-death experiences: perception is reality		**+**		
Walker et al. (1991)	Three near-death experiences with premonitions of what could have been	**+**	**+**	**+**	**+**
Ring (1991)	Amazing grace: the near-death experience as a compensatory gift	**+**	**+**	**+**	**+**
Zhi-Ying and Jian-Xun (1992)	Near-death experiences among survivors of the 1976 Tangshan Earthquake	**+**	**+**	**+**	**+**
Blackmore (1993)	Near-Death Experiences in India: They Have Tunnels Too	**+**	**+**	**+**	
Schoenbeck (1993)	Exploring the mystery of near-death experiences		**+**	**+**	**+**
Greyson (1997)	The near-death experience as a focus of clinical attention	**+**	**+**	**+**	**+**
Ring and Cooper (1997)	Near-death and out-of-body experiences in the blind: a study of apparent eyeless vision	**+**	**+**	**+**	**+**
Murphy (2001)	Near-death experiences in Thailand		**+**		**+**
Knoblauch et al. (2001)	Different kinds of near-death experience: a report on a survey of near-death experiences in Germany	**+**	**+**	**+**	**+**
Van Lommel et al. (2001)	Near-death experience in survivors of cardiac arrest: a prospective study in the Netherlands	**+**	**+**	**+**	**+**
Pasricha (1993)	Near-death experiences in South India: a Systematic survey		**+**		**+**
Purkayastha and Mukherjee (2012)	Three cases of near-death experience: is it physiology, physics or philosophy?	**+**	**+**	**+**	
Ghasemiannejad et al. (2014)	Iranian Shiite Muslim near-death experiences: features and aftereffects including dispositional gratitude	**+**	**+**	**+**	**+**
Ghasemiannejad-Jahromi et al. (2018)	The Investigation of near-death experiences, and necessity of awareness about its elements	**+**	**+**	**+**	**+**
Thomas (2020)	A near-death experience: a surgeon's validation	**+**	**+**	**+**	**+**
Panagore (2020)	My deaths direct my life: living with near-death experience	**+**	**+**	**+**	**+**
Boado et al. (2020)	A case study on near death experience, its perceived effects and coping mechanisms: input to psychological and emotional adjustment training program		**+**		**+**
Khoshab et al. (2020)	Near-death experience among Iranian Muslim cardiopulmonary resuscitation survivors	**+**	**+**	**+**	**+**

### Methodological quality

Despite all the differences in methodological design and quality, none of the 21 studies received more than 5 negative ratings; therefore, they were all included (Tables 5, 6). All the studies clearly described their research objectives, used an appropriate research methodology and design, and collected data in a way that answered the research question. In addition, Table 2 examines the quality of studies; 11 studies were of poor quality, 37 of medium quality, and 5 of good quality.

**Table 5:** Quality assessment for case study/case series that we include in this article.

**Study title**	**Question 1**	**Question 2**	**Question 3**	**Question 4**	**Question 5**	**Question 6**	**Question 7**	**Question 8**	**Question 9**	**Question 10**	**Total qualityassessment score**
The near-death experiences- is the presence always the higher self	No	Yes	Unclear	No	Unclear	Yes	Yes	Not applicable	No	Yes	4/10
2 native American near-death experiences	Unclear	Yes	Not applicable	Not applicable	YES	No	No	Not applicable	No	No	2/10
Near-death experiences in the very young	No	Unclear	Unclear	No	No	Yes	Yes	Yes	No	Yes	4/10
near-death experiences in India- a preliminary report	Yes	Unclear	Yes	No	No	Yes	Yes	Not applicable	No	Yes	5/10
The investigation of near-death experiences, and necessity of awareness about its elements	Yes	Yes	Yes	Yes	Yes	No	No	Not applicable	Yes	Yes	7/10
An Israeli account of a near-death experience: a case study of cultural dissonance	No	Yes	Yes	Not applicable	Unclear	Yes	Yes	Unclear	No	Yes	5/10
The devil in heaven: a near-death experience with both positive and negative facets	No	Yes	Unclear	No	Unclear	No	Yes	No	Unclear	Unclear	2/10
Near-death experiences and self-transformation	Yes	Yes	Not applicable	Not applicable	Not applicable	Yes	Yes	Yes	Yes	Yes	7/10
Three cases of near-death experience: is it physiology, physics or philosophy?	No	Yes	Yes	Not applicable	Unclear	Yes	Yes	No	No	Yes	5/10
A near-death experience in a 7-year-old child	No	Yes	No	Not applicable	Yes	Yes	Yes	Unclear	Yes	Yes	6/10
Near-death-like experiences without life-threatening conditions or brain disorders: a hypothesis from a case report	No	Yes	Yes	Not applicable	No	Yes	Yes	Yes	Unclear	Yes	6/10
Near-death experience in a boy undergoing uneventful elective surgery under general anesthesia	No	Yes	Yes	Not applicable	No	Yes	Yes	Unclear	Unclear	Yes	5/10
Verified account of near-death experience in a physician who survived cardiac arrest	Yes	Yes	Yes	Not applicable	No	Yes	Yes	Unclear	Unclear	Yes	6/10
Out-of-body experience during awake craniotomy	No	Yes	Yes	Not applicable	No	Yes	Yes	Unclear	Yes	No	5/10
Can experiences near death furnish evidence of life after death?	No	Yes	Yes	Not applicable	No	Yes	Yes	Unclear	Unclear	Yes	5/10
Phenomenology of near-death experiences: an analysis of a Maori case study	No	Yes	Unclear	Unclear	Unclear	Yes	No	Yes	Unclear	Yes	4/10
Getting comfortable with near-death experiences. my unimaginable journey: a physician's near-death experience	No	Yes	Yes	Not applicable	No	Yes	Yes	Unclear	Yes	Yes	6/10
A near-death experience: a surgeon's validation	No	Yes	Unclear	Not applicable	No	Yes	Yes	No	No	No	3/10
My deaths direct my life: living with near-death experience	No	Yes	Yes	Not applicable	No	Yes	Yes	Yes	No	Yes	6/10
An unanticipated cardiac arrest and unusual post resuscitation psycho-behavioral phenomena/ near death experience in a patient with pregnancy induced hypertension and twin pregnancy undergoing elective lower segment cesarean section	No	Yes	Unclear	Not applicable	No	Yes	Yes	Yes	Yes	Yes	6/10
Near-death experience by proxy: a case study	No	Yes	Unclear	Unclear	Unclear	Yes	No	Yes	Unclear	Yes	4/10
The near-death experience of a non-verbal person with congenital quadriplegia	No	Yes	Unclear	No	Unclear	Yes	Yes	No	Unclear	Unclear	3/10
Out-of-body experiences in the blind	Yes	Yes	Yes	Yes	No	Yes	Yes	Yes	Unclear	Yes	8/10
Pediatric near-death experiences	Yes	Yes	Yes	Unclear	Yes	Yes	Yes	Yes	Yes	Yes	9/10
Near-death experiences: perception is reality	No	Unclear	Yes	Unclear	No	No	No	No	Unclear	Unclear	1/10
Three near-death experiences with premonitions of what could have been	No	Yes	Unclear	No	Unclear	Yes	Yes	No	Unclear	Unclear	3/10
Near-death experiences in a pediatric population- a preliminary report	Yes	Yes	Unclear	Yes	Yes	Yes	Yes	No	No	Yes	7/10
A physician's near-death experience	No	Unclear	Yes	Unclear	Unclear	Yes	Yes	No	No	No	2/10
Exploring the mystery of near-death experiences	No	No	Yes	Unclear	Unclear	No	Yes	No	No	No	2/10
Amazing grace: the near-death experience as a compensatory gift	Yes	Yes	Unclear	No	Unclear	No	Yes	No	No	No	3/10
Near-death experiences in india: they have tunnels too	Yes	Yes	Yes	Yes	Yes	Unclear	Yes	Yes	Yes	Yes	9/10
A near-death experience among the Mapuche people	No	Unclear	Unclear	Unclear	Unclear	No	No	No	No	No	0/10
A near-death experience followed by the visitation of an “angel-like” being	Not applicable	Not applicable	Not applicable	Not applicable	Not applicable	Yes	Unclear	Yes	Yes	Yes	4/10
Near-death experiences in South India: a Systematic survey	Yes	Yes	Unclear	Yes	Yes	Yes	Unclear	No	Yes	Yes	7/10
Different kinds of near-death Experience: a report on a survey of near-death experiences in Germany	Yes	Yes	Yes	Yes	Yes	Unclear	No	Unclear	Yes	Yes	7/10
A Hawaiian near-death experience	No	Yes	Unclear	No	Unclear	No	Yes	No	No	Yes	3/10
The near-death experience as a shamanic initiation: a case study	Yes	Yes	Yes	Not applicable	Not applicable	Yes	Yes	No	No	Yes	6/10
A child's encounter with the devil: an unusual near-death experience with both blissful and frightening elements	Not applicable	Not applicable	yes	Not applicable	Not applicable	Yes	Yes	Yes	Yes	Yes	6/10
Near-death experience and out of body phenomenon during torture–a case report	No	Yes	Yes	Not applicable	Unclear	Yes	Yes	No	Yes	Yes	6/10
A near-death experience at birth	Yes	Yes	Unclear	Not applicable	Unclear	Yes	Yes	Yes	No	Yes	6/10
Recurrent near-death experience with post-vagotomy syndrome	No	Yes	Yes	Not applicable	Unclear	Yes	Yes	No	Yes	Yes	6/10
Full neurological recovery from *Escherichia coli* meningitis associated with near-death experience	Not applicable	Yes	Yes	Not applicable	Unclear	Yes	Yes	Yes	Yes	Yes	7/10
[Migraine with multiple visual symptoms and out-of-body experience may mimic epilepsy]	Not applicable	Yes	Yes	Not applicable	Unclear	Yes	Yes	Unclear	Yes	Yes	6/10
Out-of-body experience during awake craniotomy	Not applicable	Yes	Yes	Not applicable	Unclear	Yes	Yes	No	Yes	Yes	6/10
[Out-of-body experience as possible seizure symptom in a patient with a right parietal lesion]	Not applicable	Yes	Unclear	Not applicable	Unclear	Yes	Yes	Unclear	Yes	Yes	5/10
Out-of-body experience and auditory and visual hallucinations in a patient with cardiogenic syncope: crucial role of cardiac event recorder in establishing the diagnosis	Not applicable	Yes	Unclear	Not applicable	Unclear	Yes	Yes	Yes	Yes	Yes	6/10
The near-death experience as a focus of clinical attention	Yes	Yes	Yes	Unclear	Unclear	Yes	Yes	No	Unclear	Yes	6/10

Answer only with: yes/ no/ unclear/not applicable.

**Table 6:** Quality assessment for qualitative study that we include in this article.

**Study title**	**Question 1**	**Question 2**	**Question 3**	**Question 4**	**Question 5**	**Question 6**	**Question 7**	**Question 8**	**Question 9**	**Question 10**	**Total qualityassessment score**
Near-death experiences in a Pacific Northwest American population: the Evergreen study. Anabiosis	Yes	Yes	Yes	Unclear	Unclear	Unclear	Yes	Yes	Yes	Yes	7/10
A case Study on near death experience, its perceived effects and coping mechanisms: input to psychological and emotional adjustment training program	Yes	Yes	Yes	No	Yes	Unclear	No	No	Yes	Yes	6/10
Near-death and out-of-body experiences in the blind: a study of apparent eyeless vision	Yes	Yes	Yes	Yes	Yes	Yes	Yes	Yes	Yes	Yes	10/10
Near-death experience in survivors of cardiac arrest: a prospective study in the Netherlands	Yes	Yes	Yes	Yes	Yes	Unclear	No	No	Yes	Yes	7/10
Near-death experiences among survivors of the 1976 Tangshan earthquake	Yes	No	Yes	Yes	Yes	Unclear	No	Yes	Yes	Yes	7/10
Near-death experience among Iranian Muslim cardiopulmonary resuscitation survivors	Yes	Unclear	Yes	Yes	Yes	Unclear	No	Yes	Yes	Yes	7/10
Iranian Shiite Muslim near-death experiences: features and aftereffects including dispositional gratitude	Yes	Yes	Yes	Yes	Yes	Unclear	No	Yes	Yes	Yes	8/10

## Discussion

This systematic revue study was conducted with the aim of explaining Individuals' near-death experiences and identifying common experiences. The results of this study are categorized into 4 main categories including emotional, cognitive, religious, spiritual and supernatural experiences.

Supernatural experiences were the most frequent category of experiences related to NDE, which consist of two subcategories: out of body experiences, and supernatural and metaphysical perceptions. In many studies, supernatural perceptions include passing through a tunnel involuntarily, moving toward the ceiling [out-of-body experience (OBE)], seeing one's own physical body from above whilst outside the body (the phenomenon of self-bilocation), having awareness of the places far from the body, self-permeability (passing through physical objects such as walls), being present in several locations at the same time (self-multilocation) (composed bodies), the feeling of being floating, entering a non-terrestrial location (heaven), and telepathy (non-verbal communication) with others. It can be said that the most important feature of NDEs is an out-of-body experience (OBE), which had been experienced by the majority of the NDErs. OBE is a type of autoscopy (literally, “watching oneself”) in which the soul is separated from the body, but the individual is in a fully conscious state or beyond normal consciousness (Long and Perry, 2010). Soul, in religion and philosophy, the immaterial aspect or essence of a human being that which confers individuality and humanity, often considered to be synonymous with the mind or the self. For most theologies, the Soul is further defined as that part of the individual, which partakes of divinity and transcends the body in different explanations (Ciocan, 2019). The individual seems to be awake, and watches his body and the world from a disembodied place and outside his physical body (Blanke et al., 2016), or perceives verified events that have occurred at a distance outside his/her scope (Greyson et al., 2009). A typical narrative is: “I was lying on the bed. Suddenly, I ascended in a suspended state, watching myself and the events that were taking place from somewhere above the floor, for example, near the ceiling.” (Green, 1968; Van Lommel, 2010). After an OBE, some individuals have had numerous supernatural perceptions. In some cases, there is a higher number of perceived experiences and, in others, there are fewer ones. However, there are many commonalities among the mentioned metaphysical experiences. The results of various studies show that after the soul leaves the body, NDErs enter a cylindrical tunnel, at the beginning of which there is absolute darkness and, at the end, a very dazzling light toward which the individual is guided. In most cases, this experience has been a very difficult one to forget. This experience is called a Tunnel Experience (TE) (Sabom, 1978). A tunnel experience may be defined as the perception of a realistic enclosed space which is much longer than its diameter. The peripheral features and the deep perspectives of this phenomenon indicate organizing the space around a central area in the visual field (Moody, 2001). Tunnel experiences have been reported in different forms including cylinder, pipe, tunnel, passage, corridor, spiral, well, funnel, shaft, hole, culvert, cave, long enclosure, sewer, cone, and so on (Drab, 1981). Greyson claims that crossing the tunnel occurs mostly for Indian and Buddhist NDEr's (Greyson, 2015). However, the results of another study conducted on the Muslim population have also confirmed similar experiences (Ghasemiannejad-Jahromi A and R., 2018). In his study, Todd Murphy states that tunnels are not seen or are very rare in Thai NDEs (Murphy, 2001). This systematic review study shows that the supernatural and metaphysical experiences of the participants have similar roots for every race and religion, with differences in the expression of details.

The second category of NDEs were spiritual and religious experiences, consisting of the 4 subcategories: meeting with the dead and acquaintances, meeting with religious figures, feeling oneness with the universe, and observing punishment and reward for actions. Some NDEr's have reported encounters with their deceased relatives and friends (Tassell-Matamua, 2013; Ghasemiannejad et al., 2014). Additionally, some children who have experienced NDEs have reported meeting the individuals whom they did not know at the time of the NDE, but later recognized as their deceased relatives from the family photos they had never seen before (Morse et al., 1985; Lopez et al., 2006). Other NDErs report encountering a recently deceased individual, whose death they hadn't been aware of (Greyson, 2010). One of the common aspects of the experiences was meeting with religious figures. The results of a study that compared NDE experiences in different cultures show that in western NDEs, when one is in the tunnel, he/she perceives that a group of deceased relatives and friends have come to welcome him/her, while in Thai NDEs, the experiences usually start with Yamatoots (Yamadutas are the messengers of death according to Hinduism, the agents of Yama, the god of the netherworld) (Murphy, 2001). In Thai NDEs, there is no experience of *being light*, and the Buddha appears only symbolically. One of the Thai experienced mentioned, “I asked [Yamatoot] to take me to visit the Lord Buddha. I told him I had to see the Buddha. Yamatoot looked up and pointed at the sky, saying, ‘That big star is the Buddha”' (Moody, 2001). In Western NDEs, the majority of the NDErs were Christians, and had seen the figures associated with Jesus Christ and the apostles (Greyson, 2010). In a study whose target population were Twelver Shīʿīsm (also known as Imāmīyyah, is the largest branch of Shīʿa Islam, comprising about 85 percent of all Shīʿa Muslims), the reported religious and spiritual figures were among Shiite imams (Ghasemiannejad-Jahromi et al., 2018). For a better understanding of this category, the individuals' religious and cultural backgrounds should be considered while interpreting the experiences, encounters, and observations. The results of some other studies reveal a feeling of oneness with the universe and the whole cosmos, where the NDErs had stated that they had unified with the whole universe or a part of creation such as plants, with no distance between them (Long and Perry, 2010; Ghasemiannejad et al., 2014). The idea that the individual is inextricably connected to the rest of the world, or that everything is part of a whole, can be found in many of the world's religious, spiritual, and philosophical traditions (Ivanhoe et al., 2018). Most of the individuals who have experienced the feeling of oneness say that they will choose this state of mind if they have eternal life. Oneness is perhaps the deepest and the most sublime state that a human being can achieve (Klussman, 2022). The last subcategory of spiritual-religious experiences was observing punishment and reward for actions. Research shows that some NDEr's are able to perceive the external consequences of their actions and deeds in the world, as well as their inner and hidden effects (Holden et al., 2009; Greyson, 2014; Khanna and Greyson, 2014b).

Another category of NDEs were cognitive experiences, which consisted of 4 subcategories, including heightened senses, an altered nature of time, reviewing life events, and the sudden perception of a specific knowledge. In regard with the heightened senses, a review of the reports of the NDEr's shows that their visual descriptions are impressive (efficacious) and clear (obvious), all while these individuals are unconscious and often clinically dead at the time of experiencing and seeing such wonderful sights. In NDEs, all the senses of sight, hearing, touch, taste, and smell have been described. The heightened senses and the improved consciousness among these individuals even indicate that these experiences are to be very different from dreams and sleep, and at the moment it is difficult to find a recognized medical explanation for NDEs. This phenomenon is medically inexplicable. There is no other type of altered consciousness experience in which events are that clear, consciousness is that strong, and events follow one another in such a specific order. The research conducted in this field shows a stable pattern of enhanced consciousness and heightened senses, which leads to the clarity of NDEs and proves them (Bryant and Peck, 2009; Khanna and Greyson, 2014b). Moreover, according to some experienced, in NDEs, time loses its meaning and sense, and they see the events of their life in a fraction of a second (Holden et al., 2009). Reviewing the past events of one's life is another cognitive experience in which NDErs may see a part or all of their life. The individual's encounter with *self* is one of the most important and common features of these experiences. At this stage, one encounters his/her own words, actions, and thoughts, and sees his/her own life in the form of a book, show, or movie, and judges it. The results of other research show that while reviewing their lives, the individuals review their past actions, words, and thoughts, and realize that each of them has a special energy, which has affected both themselves and others in this world (Facco and Agrillo, 2012b; Tassell-Matamua, 2014). In addition, the results of various studies, including Long's research, state that the events observed in the NDEr's life reviews are based on reality. These results assume that if NDEs are real, it is expected that the events observed during the life review be confirmed by the individual, and vice versa, if NDEs are not real, significant errors must occur during the life review. However, the latter is not the case, and everything has been confirmed by the individuals (Bryant and Peck, 2009; Khanna and Greyson, 2014b).

The last category of near-death experiences is emotional experiences, which includes two subcategories: positive experiences and negative experiences. Many NDErs state that they have experienced immense peace, and that it has been their most memorable experience, in such a way that they hesitated whether or not to return to life. In addition, in the cases where an individual had died with severe pain, his/her pain had disappeared with the sudden experience of relaxation (Klemenc-Ketis et al., 2010; Long and Perry, 2010). Most of the early studies on NDEs depicted only positive emotions (Ring, 1980, 1984). However, an interdisciplinary study was published, in which they identified 55 NDErs, eleven of whom reported negative experiences (Lindley et al., 1981). Another study indicated that 1–10% of the samples had not described positive feelings (Charland-Verville et al., 2014), these different proportions can be attributed to very broad definitions of disturbing NDEs, as well as different methods (Greyson, 2003; Charland-Verville et al., 2014). Reviewing the conducted studies shows that hellish and purgatory scenes are rarely found in NDEs, but heavenly scenes are seen more often and are very similar to each other. It may be concluded that the disturbing dimensions of the experience, added to its mystical aspect, can prevent the individuals from sharing it (Cassol et al., 2019). Based on the results of a study, frightening NDEs are divided into three groups: 1. The negative events may be viewed as warnings about unwise actions, leading to self-analysis and, ultimately, a “spin” in the NDEr's life, 2. The NDEr may treat the event as if it is not important, and 3. The frightening event may lead to difficulty in integrating the experience, developing a sense of stigma (Greyson, 2014).

## Advantages and limitations

The present study has combined data focusing on the principle of comprehensiveness and quality. To perform a comprehensive search, the synonym recognition systems of Thesaurus Mesh and Emtree were used to determine the keywords. Then the search was done in the vastest electronic databases such as PubMed, Scopus, Web of Science, and ProQuest with a wide time range, using experts' opinions, without time or place limitations. Considering the variety of the experiences reported by the experienced, the results were reported qualitatively. Some of the studies date back to the years before 1990, some of whose PDFs were incomplete, and the data of a number of their cases had been presented incompletely.

## Conclusion

It can be almost concluded that according to the researchers who have presented valuable research in this field, the basis and the content of the patterns mentioned by the NDEr's are similar, and the differences are in the explanation and the interpretation of the experience. There is a common core among them such as out-of-body experiences, passing through a tunnel, heightened senses, etc. This is what all ethnic groups and nations face, without exception and without being influenced by religion, race, culture, and the native customs of their countries. Besides this central core, a series of other events or actions take place, which are more detailed and rooted in the personal archive of the NDEr's, consisting of all kinds of symbols, images, and characters which have been important only to that person. It is clear that aspects of near-death experiences are influenced by culture, while there are also parts that are universal. The most critical versatile features include altered states of consciousness and delusions, which seem to occur in all cultures studied so far. However, the specific characteristics of this experience vary significantly according to cultural context. The first point is that apparently, the content of experiences shows variations. For example, in some cultures, certain religious figures may be seen, and unlike them, others may see their deceased relatives. Second, the pattern of this experience is diverse, so people from certain cultures may have the experience of leaving the body, going to the tunnel, and reviewing life, and unlike them, the experience of others does not include these, and finally, the concept and perception of the near-death experience are different among cultures. In the current study, four main NDE categories were extracted from case reports, case series, and qualitative research studies, in the majority of which the experiences were common. The heightened senses and the improved consciousness among these individuals even indicate that “these experiences are neither dreams, nor sleep, nor the disorders caused”; “This phenomenon is medically inexplicable.” The research conducted in this field show a stable pattern of enhanced consciousness and heightened senses, “which leads to the clarity of NDEs and proves their being real.” The familiarity of the treatment staff, especially nurses and doctors, with NDE components and elements, gaining knowledge in this regard, and an awareness of appropriate and pertinent interventions can lead to proper reactions and feedbacks in response to the NDEr.

## Data availability statement

The original contributions presented in the study are included in the article/supplementary material, further inquiries can be directed to the corresponding author.

## Author contributions

HA, AH, AO, and MR designed the study, supervised and directed the study, carried out the implementation, aided in designing the study, and worked on the manuscript. HA and AH processed the experimental data, performed the analysis, and drafted the manuscript. All authors discussed the results, commented on the manuscript, and approved the final manuscript.

## References

[B1] Abramovitch H. (1988). An Israeli account of a near-death experience: a case study of cultural dissonance. J. Near Death Stud. 6, 175–184. 10.1007/BF01073366

[B2] Bhowmick B. K. (1991). Recurrent near-death experience with post-vagotomy syndrome. J. R. Soc. Med. 84, 311. 10.1177/014107689108400523 2041015 PMC1293237

[B3] Blackmore S. J. (1993). Near-death experiences in India: they have tunnels too. J. Near Death Stud. 11, 205–217. 10.1007/BF01078238

[B4] Blanke O. Faivre N. Dieguez S. (2016). “Chapter 20 - leaving body and life behind: out-of-body and near-death experience,” in The Neurology of Conciousness, 2nd Edn., eds S. Laureys, O. Gosseries, and G. Tononi (San Diego, CA: Academic Press), 323–347. 10.1016/B978-0-12-800948-2.00020-0

[B5] Blanke O. Faivre N. Dieguez S. Laureys S. Gosseries O. Tononi G. (2009). The Neurology of Consciousness. London: Academic Publishers.

[B6] Boado J. A. V. Kilala A. M. C. Niduaza J. C. Padilla D. D. M. (2020). A case study on near death experience, its perceived effects and coping mechanisms: input to psychological and emotional adjustment training program. Ann. Med. Psychol. 178, 535–539. 10.1016/j.amp.2019.05.001

[B7] Bonenfant R. J. (2000). A near-death experience followed by the visitation of an “angel-like” being. J. Near Death Stud. 19, 103–113. 10.1023/A:1007861022420

[B8] Bonenfant R. J. (2001). A child's encounter with the devil: an unusual near-death experience with both blissful and frightening elements. J. Near Death Stud. 20, 87–100. 10.1023/A:1013058221999

[B9] Bos E. M. Spoor J. K. H. Smits M. Schouten J. W. Vincent A. (2016). Out-of-body experience during awake craniotomy. World Neurosurg. 92, 586.e589–586.e513. 10.1016/j.wneu.2016.05.002 27178238

[B10] Brandt C. Brechtelsbauer D. Bien C. G. Reiners K. (2005). [Out-of-body experience as possible seizure symptom in a patient with a right parietal lesion]. Nervenarzt 76, 1259, 1261–1252. 10.1007/s00115-005-1904-y 15830175

[B11] Brandt C. Kramme C. Storm H. Pohlmann-Eden B. (2009). Out-of-body experience and auditory and visual hallucinations in a patient with cardiogenic syncope: crucial role of cardiac event recorder in establishing the diagnosis. Epilepsy Behav. 15, 254–255. 10.1016/j.yebeh.2009.02.047 19268716

[B12] Bryant C. D. Peck D. L. (2009). Encyclopedia of Death and the Human Experience. Sage. 10.4135/978141297203134414947

[B13] Campbell R. Pound P. Morgan M. Daker-White G. Britten N. Pill R. . (2012). Evaluating meta ethnography: systematic analysis and synthesis of qualitative research. Health Technol Assess. 15, 1–164 10.3310/hta15430 22176717

[B14] Cassol H. Martial C. Annen J. Martens G. Charland-Verville V. Majerus S. . (2019). A systematic analysis of distressing near-death experience accounts. Memory 27, 1122–1129. 10.1080/09658211.2019.1626438 31189413

[B15] Charland-Verville V. Jourdan J.-P. Thonnard M. Ledoux D. Donneau A.-F. Quertemont E. . (2014). Near-death experiences in non-life-threatening events and coma of different etiologies. Front. Hum. Neurosci. 8, 203. 10.3389/fnhum.2014.00203 24904345 PMC4034153

[B16] Ciocan C. T. (2019). The value of the soul in the religious views. An overview targeting the salvation of an individual. Dialogo 6, 233–244. 10.18638/dialogo.2020.6.2.21

[B17] Cooper M. J. (2011). Near-death experience and out of body phenomenon during torture–a case report. Torture. 21, 178–181. 22057105

[B18] Drab K. J. (1981). The tunnel experience: reality or hallucination? Anabiosis J. Near Death Stud. 1, 126–128. 10.17514/JNDS-1981:1(2)

[B19] Facco E. Agrillo C. (2012a). Near-death-like experiences without life-threatening conditions or brain disorders: a hypothesis from a case report. Front. Psychol. 3, 490. 10.3389/fpsyg.2012.00490 23162522 PMC3498963

[B20] Facco E. Agrillo C. (2012b). Near-death experiences between science and prejudice. Front. Hum. Neurosci. 6, 209. 10.3389/fnhum.2012.00209 22826697 PMC3399124

[B21] Foster R. D. James D. Holden J. M. (2009). “Practical applications of research on near-death experiences,” in The Handbook of Near-Death Experiences: Thirty Years of Investigation, eds J. M. Holden, B. Greyson, and D. James (Praeger/ABC-CLIO), 235–258.

[B22] Gagnier J. J. Kienle G. Altman D. G. Moher D. Sox H. Riley D. (2013). The CARE guidelines: consensus-based clinical case reporting guideline development. BMJ Case Rep. 2013, bcr2013201554. 10.1136/bcr-2013-201554 24228906 PMC3844611

[B23] Ghasemiannejad A. Long J. Nouri F. F. Krahnakian K. (2014). Iranian shiite muslim near-death experiences: features and aftereffects including dispositional gratitude. J. Near Death Stud. 33, 30–42. 10.17514/JNDS-2014-33-1-p30-42

[B24] Ghasemiannejad-Jahromi A. Mehrabizadeh-Honarmand M. Hashemi S. Beshlideh K. Khojasteh-Mehr R. (2018). The investigation of near-death experiences, and necessity of awareness about its elements. J Qualit. Res. Health Sci. 7, 337–348. Available online at: https://www.magiran.com/paper/19269299173662

[B25] Gómez-Jeria J. S. (1993). A near-death experience among the Mapuche people. J. Near Death Stud. 11, 219–222. 10.1007/BF01078239

[B26] Graneheim U. H. Lundman B. (2004). Qualitative content analysis in nursing research: concepts, procedures and measures to achieve trustworthiness. Nurse Educ. Today 24, 105–112. 10.1016/j.nedt.2003.10.001 14769454

[B27] Green C. E. (1968). Out-of-the-Body Experiences. Institute of Psychophysical Research.

[B28] Green J. T. (2001). The near-death experience as a shamanic initiation: a case study. J. Near Death Stud. 19, 209–225. 10.1023/A:1007859024038

[B29] Greyson B. (1983). The near-death experience scale. J. Nervous Mental Dis. 171, 369–375. 10.1097/00005053-198306000-00007 6854303

[B30] Greyson B. (1997). The near-death experience as a focus of clinical attention. J. Nerv. Ment. Dis. 185, 327–334. 10.1097/00005053-199705000-00007 9171810

[B31] Greyson B. (2003). Incidence and correlates of near-death experiences in a cardiac care unit. Gen. Hosp. Psychiatry 25, 269–276. 10.1016/S0163-8343(03)00042-2 12850659

[B32] Greyson B. (2007). Consistency of near-death experience accounts over two decades: are reports embellished over time? Resuscitation 73, 407–411. 10.1016/j.resuscitation.2006.10.013 17289247

[B33] Greyson B. (2010). Seeing dead people not known to have died: “Peak in Darien” experiences. Anthropol. Hum. 35, 159–171. 10.1111/j.1548-1409.2010.01064.x

[B34] Greyson B. (2014). “Near-death experiences,” in Varieties of Anomalous Experience: Examining the Scientific Evidence., eds E. Cardeña, S. J. Lynn, and S. Krippner (Washington, DC: American Psychological Association), 312–352. 10.1037/14258-012

[B35] Greyson B. (2015). Western scientific approaches to near-death experiences. Humanities 4, 775–796. 10.3390/h4040775

[B36] Greyson B. Holden J. M. James D. (2009). The Handbook of Near-Death Experiences: Thirty Years of Investigation: Thirty Years of Investigation. United America: Praeger. 1, 316.

[B37] Groth-Marnat G. Summers R. (1998). Altered beliefs, attitudes, and behaviors following near-death experiences. J. Hum. Psychol. 38, 110–125. 10.1177/00221678980383005

[B38] Hausheer J. R. (2014). Getting comfortable with near-death experiences. My unimaginable journey: a physician's near-death experience. Mo. Med. 111, 180–183 25011329 PMC6179563

[B39] Hausheer J. R. (2020). A physician's near-death experience. Narrat. Inquiry Bioeth. 10, 11–14. 10.1353/nib.2020.0001 33416536

[B40] Herzog D. B. Herrin J. T. (1985). Near-death experiences in the very young. Crit. Care Med. 13, 1074–1075. 10.1097/00003246-198512000-00021 4064720

[B41] Hess G. (2019). Physicalism, supernaturalism, and near-death experiences: A phenomenological perspective. J. Consciousness Stud. Imprint Academic. 26, 86–106.

[B42] Holden J. M. Long J. Maclurg B. J. (2009). “Characteristics of Western near-death experiencers,” in The Handbook of Near-Death Experiences: Thirty Years of Investigation, ed E. D. James (American Psychological Association), 109–133. Available online at: https://psycnet.apa.org/record/2009-13429-000

[B43] Hosokawa K. Usami K. Kajikawa S. Shimotake A. Tatsuoka Y. Ikeda A. . (2021). [Migraine with multiple visual symptoms and out-of-body experience may mimic epilepsy]. Rinsho Shinkeigaku 61, 530–536. 10.5692/clinicalneurol.cn-001577 34275950

[B44] Hsieh H.-F. Shannon S. E. (2005). Three approaches to qualitative content analysis. Qual. Health Res. 15, 1277–1288. 10.1177/1049732305276687 16204405

[B45] Institute of Medicine (US) Committee on Standards for Systematic Reviews of Comparative Effectiveness Research. (2011). Finding What Works in Health Care: Standards for Systematic Reviews. Eden J, Levit L, Berg A, Morton S, editors. Washington, DC: National Academies Press. 24983062

[B46] Irwin H. J. (1987). Out-of-body experiences in the blind. J. Near-Death Stud. 6, 53–60. 10.1007/BF01073268

[B47] Irwin H. J. Bramwell B. A. (1988). The devil in heaven: a near-death experience with both positive and negative facets. J. Near-Death Stud. 7, 38–43. 10.1007/BF01076748

[B48] Ivanhoe P. Flanagan O. Harrison V. Schwitzgebel E. Sarkissian H. (2018). The Oneness Hypothesis: Beyond the Boundary of Self . Columbia University Press.

[B49] Kellehear A. (2001). An Hawaiian near-death experience. J. Near Death Stud. 20, 31–35. 10.1023/A:1011164711148

[B50] Kelly E. W. Greyson B. Stevenson I. (2000). Can experiences near death furnish evidence of life after death? Omega 40, 513–519. 10.2190/KNTM-6R07-LTVT-MC6K

[B51] Khanna S. Greyson B. (2014a). Daily spiritual experiences before and after near-death experiences. Psychol. Relig. Spiritual. 6, 302. 10.1037/a0037258 23625172

[B52] Khanna S. Greyson B. (2014b). Near-death experiences and spiritual well-being. J. Relig. Health 53, 1605–1615. 10.1007/s10943-013-9723-0 23625172

[B53] Khanna S. Moore L. E. Greyson B. (2018). Full neurological recovery from escherichia coli meningitis associated with near-death experience. J. Nerv. Ment. Dis. 206, 744–747. 10.1097/NMD.0000000000000874 30124575

[B54] Khoshab H. Seyedbagheri S. Iranmanesh S. Shahrbabaki P. M. Dehghan M. Tirgari B. . (2020). Near-death experience among iranian muslim cardiopulmonary resuscitation survivors. Iran. J. Nurs. Midwifery Res. 25, 414–418. 10.4103/ijnmr.IJNMR_190_19 33344213 PMC7737831

[B55] Klemenc-Ketis Z. Kersnik J. Grmec S. (2010). The effect of carbon dioxide on near-death experiences in out-of-hospital cardiac arrest survivors: a prospective observational study. Critical Care 14, 1–7. 10.1186/cc8952 20377847 PMC2887177

[B56] Klussman K. (2022). Find The Meaning of Connection Through Oneness.

[B57] Knoblauch H. Schmied I. Schnettler B. (2001). Different kinds of near-death experience: a report on a survey of near-death experiences in Germany. Journal of Near-Death Studies 20, 15–29. 10.1023/A:1011112727078

[B58] Lindley J. H. Bryan S. Conley B. (1981). Near-death experiences in a Pacific Northwest American population: The Evergreen study. Anabiosis J Near Death Stud. 1:106–110. Available online at: https://digital.library.unt.edu/ark:/67531/metadc799156/m1/3/

[B59] Long M. Perry P. (2010). Evidence of the Afterlife. Louisiana: Harper Collins Publishers.

[B60] Lopez U. Forster A. Annoni J. M. Habre W. Iselin-Chaves I. A. (2006). Near-death experience in a boy undergoing uneventful elective surgery under general anesthesia. Pediatr. Anesth. 16, 85–88. 10.1111/j.1460-9592.2005.01607.x 16409537

[B61] Martial C. Cassol H. Antonopoulos G. Charlier T. Heros J. Donneau A. F. . (2017). Temporality of features in near-death experience narratives. Front. Hum. Neurosci. 11, 311. 10.3389/fnhum.2017.00311 28659779 PMC5469194

[B62] Mcarthur A. Klugárová J. Yan H. Florescu S. (2015). Innovations in the systematic review of text and opinion. JBI Evid. Implement, 13, 188–195. 10.1097/XEB.0000000000000060 26207851

[B63] Moody R. A. (2001). Life after life. Mockingbird Books. 175.

[B64] Moody R. A. (2005). The Light Beyond. Bantam: Random House. 224.

[B65] Moola S. Munn Z. Tufanaru C. Aromataris E. Sears K. Sfetcu R. . (2020). “Systematic reviews of etiology and risk,” in JBI Manual for Evidence Synthesis, eds E. Aromataris and Z. Munn (JBI). 10.46658/JBIMES-20-08

[B66] Morse M. (1983). A near-death experience in a 7-year-old child. Am. J. Dis. Child. 137, 959–961. 10.1001/archpedi.1983.02140360023008 6613940

[B67] Morse M. (2013). “Parting visions: a new scientific paradigm,” in The Near-Death Experience (Routledge).

[B68] Morse M. Conner D. Tyler D. (1985). Near-death experiences in a pediatric population - a preliminary-report. Am. J. Dis. Child. 139, 595–600. 10.1001/archpedi.1985.02140080065034 4003364

[B69] Murphy T. (2001). Near-death experiences in Thailand. J. Near Death Stud. 19, 161–178. 10.1023/A:1026413705216

[B70] Noyes R. Jr. (1980). Attitude change following near-death experiences. Psychiatry 43, 234–242. 10.1080/00332747.1980.11024070 7403383

[B71] Panagore P. B. (2020). My deaths direct my life: living with near-death experience. Narrat. Inquiry Bioeth. 10, E3–E6. 10.1353/nib.2020.0008 33416525

[B72] Panditrao M. M. Singh C. Panditrao M. M. (2010). An unanticipated cardiac arrest and unusual postresuscitation psycho-behavioural phenomena/ near death experience in a patient with pregnancy induced hypertension and twin pregnancy undergoing elective lower segment caesarean section. Indian J. Anaesth. 54, 467–469. 10.4103/0019-5049.71035 21189888 PMC2991660

[B73] Parnia S. (2017). Understanding the cognitive experience of death and the near-death experience. QJM 110, 67–69. 10.1093/qjmed/hcw185 28100825

[B74] Parnia S. Waller D. G. Yeates R. Fenwick P. (2001). A qualitative and quantitative study of the incidence, features and aetiology of near death experiences in cardiac arrest survivors. Resuscitation 48, 149–156. 10.1016/S0300-9572(00)00328-2 11426476

[B75] Pasricha S. (1993). A systematic survey of near-death experiences in south India. J. Sci. Explor. 7, 161–171.

[B76] Pasricha S. Stevenson I. (1986). Near-death experiences in India a preliminary report. J. Nervous Mental Dis. 174, 165–170. 10.1097/00005053-198603000-00007 3950600

[B77] Pennachio J. (1988). Near-death experiences and self-transformation. J. Near Death Stud. 6, 162–168. 10.1007/BF01073364

[B78] Purkayastha M. Mukherjee K. K. (2012). Three cases of near death experience: is it physiology, physics or philosophy? Ann. Neurosci. 19, 104–106. 10.5214/ans.0972.7531.190303 25205979 PMC4117086

[B79] Ring K. (1980). Life at Death: A Scientific Investigation of the Near-Death Experience. Coward Mc Cann.

[B80] Ring K. (1984). Heading toward Omega: In Search of the Meaning of the Near-Death Experience. William Morrow and Company.

[B81] Ring K. (1991). Amazing grace: THE near-death experience as a compensatory gift. J. Near Death Stud. 10, 11–39. 10.1007/BF01073294

[B82] Ring K. Cooper S. (1997). Near-death and out-of-body experiences in the blind: a study of apparent eyeless vision. J. Near Death Stud. 16, 101–147. 10.1023/A:1025010015662

[B83] Sabom M. B. (1978). Physicians evaluate the near-death experience. Theta 6, 1–6. 10.1080/07481187708252891

[B84] Sabom M. B. (1982). Recollections of Death: A Medical Investigation. HarperCollins.

[B85] Schlieter J. Schlieter J. (2018). “17 The formation of near-death experiences: moody, ritchie, and hampe,” in What Is It Like To Be Dead?: Near-Death Experiences, Christianity, and the Occult (Oxford: Oxford University Press). 10.1093/oso/9780190888848.003.0003

[B86] Schnaper N. Panitz H. L. (1990). Near-death experiences: perception is reality. J. Near Death Stud. 9, 97–104. 10.1007/BF01074210

[B87] Schoenbeck S. B. (1993). Exploring the mystery of near-death experiences. Am. J. Nurs. 93, 42–46. 10.2307/3464363 8488901

[B88] Schorer C. E. (1985). 2 Native American near-death experiences. Omega J. Death Dying 16, 111–113. 10.2190/640W-8XPR-RCD5-5Y9L

[B89] Schwaninger J. Eisenberg P. R. Schechtman K. B. Weiss A. N. (2002). A prospective analysis of near-death experiences in cardiac arrest patients. J. Near Death Stud. 20, 215–232. 10.1023/A:1015258818660

[B90] Serdahely W. J. (1987). The near-death experiences is the presence always the higher self. Omega J. Death Dying 18, 129–134. 10.2190/4LAG-1UKK-C00U-BV02

[B91] Serdahely W. J. (1990). Pediatric near-death experiences. J. Near Death Stud. 9, 33–39. 10.1007/BF01074099

[B92] Serdahely W. J. Walker B. A. (1990). A near-death experience at birth. Death Stud. 14, 177–183. 10.1080/07481189008252359

[B93] Sutherland C. (1990). Near-death experience by proxy: a case study. J. Near Death Stud. 8, 241–251. 10.1007/BF01074277

[B94] Tassell-Matamua N. (2013). Brief report: phenomenology of near-death experiences: an analysis of a Māori case study. J. Near Death Stud. 32, 107–117. 10.17514/JNDS-2013-32-2-p107-117

[B95] Tassell-Matamua N. A. (2014). Near-death experiences and the psychology of death. OMEGA J. Death Dying 68, 259–277. 10.2190/OM.68.3.e 24834668

[B96] Thomas K. (2020). A near-death experience: a surgeon's validation. Narrat. Inquiry Bioeth. 10, 26–29. 10.1353/nib.2020.0013 33416543

[B97] Thonnard M. Charland-Verville V. Brédart S. Dehon H. Ledoux D. Laureys S. . (2013). Characteristics of near-death experiences memories as compared to real and imagined events memories. PLoS ONE 8, e57620. 10.1371/journal.pone.0057620 23544039 PMC3609762

[B98] Van Lommel L. (2010). Consciousness Beyond Life. HarperCollins; HarperOne.

[B99] van Lommel P. van Wees R. Meyers V. Elfferich I. (2001). Near-death experience in survivors of cardiac arrest: a prospective study in the Netherlands. Lancet. 358, 2039–2045. 10.1016/S0140-6736(01)07100-8 11755611

[B100] Van Lommel P. Van Wees R. Meyers V. Elfferich I. (2017). “Near-death experience in survivors of cardiac arrest: a prospective study in the Netherlands,” in Parapsychology (Routledge), 91–97. 10.4324/9781315247366-5 11755611

[B101] Walker B. A. Serdahely W. J. Bechtel L. J. (1991). Three near-death experiences with premonitions of what could have been. J. Near Death Stud. 9, 189–196. 10.1007/BF01074181

[B102] Williams H. L. Conway M. A. Cohen G. (2008). “Autobiographical memory,” in Memory in the Real World, 3rd Edn, eds G. Cohen and M. A. Conway (Hove: Psychology Press), 21–90.

[B103] Woollacott M. Peyton B. (2021). Verified account of near-death experience in a physician who survived cardiac arrest. Explore . 17, 213–219. 10.1016/j.explore.2020.03.005 32245708

[B104] Zhi-Ying F. Jian-Xun L. (1992). Near-death experiences among survivors of the 1976 Tangshan earthquake. J. Near Death Stud. 11, 39–48. 10.1007/BF010827361478139

